# Degradation of pharmaceuticals and other emerging pollutants employing bi-metal catalysts/magnesium and/or (green) hydrogen in aqueous solution

**DOI:** 10.1007/s11356-024-32777-1

**Published:** 2024-05-15

**Authors:** Volker Birke, Rahul Singh, Oliver Prang

**Affiliations:** Hochschule Wismar – University of Applied Sciences, Technology, Business and Design, Faculty of Engineering Science, Department of Mechanical, Process and Environmental Engineering, Philipp-Müller-Str. 14, 23966 Wismar, Germany

**Keywords:** Catalytic hydrodehalogenation and hydrogenation, Iron bimetals, Kinetic analysis, Magnesium, Pharmaceutical pollutants

## Abstract

Contaminations by pharmaceuticals, personal care products, and other emerging pollutants in water resources have become a seriously burgeoning issue of global concern in the first third of the twenty-first century. As societal reliance on pharmaceuticals continues to escalate, the inadvertent introduction of these substances into water reservoirs poses a consequential environmental threat. Therefore, the aim of this study was to investigate reductive degradation, particularly, catalytic hydrogenation regarding model pollutants such as diclofenac (DCF), ibuprofen (IBP), 17α-ethinylestradiol (EE2), or bisphenol-A (BPA), respectively,  in aqueous solutions at lab scale. Iron bimetals (zero valent iron, ZVI, and copper, Cu, or nickel, Ni) as well as zero valent magnesium (Mg, ZVM) in combination with  rhodium, Rh, or palladium, Pd, as hydrogenation catalysts (HK), were investigated. Studies were executed through various short-term batch experiments, with multiple sample collections, over a total range of 120 min. The results indicated that DCF was attenuated at over 90 % when exposed to Fe-Cu or a Fe-Ni bimetal (applied as a single model pollutant). However, when DCF was part of a mixture alongside with IBP, EE2, and BPA, the attenuation efficacy decreased to 79 % with Fe-Cu and 23 % with Fe-Ni. Conversely, both IBP and BPA exhibit notably low attenuation levels with both bimetals, less than 50 %, both deployed as single substances or in mixtures. No reaction (degradation) products could be identified employing LC-MS, but sometimes a release of the parent pollutant when applying an acetic acid buffer could be noted to a certain extent, suggesting adsorption processes on corrosion products such as iron hydroxide and/or oxides. Surprisingly, Mg in combination with Rh (Rh-HK) or Pd (Pd-HK) showed a significantly rapid decrease in the concentrations of DCF, EE2, and BPA, in part up to approximately 100 %, that is, within a few minutes only in part due to hydrogenation degradation reactions (related reaction products could actually be identified by LC-MS; adsorption processes were not observed here). Moreover, kinetic modeling of the DCF degradation with Mg-Rh-HK was conducted at different temperatures (15 °C, 20 °C, 25 °C, 35 °C) and varied initial concentrations (2.5 mg/L, 5.0 mg/L, 7.5 mg/L, 10.0 mg/L). The outcomes prove that the degradation of DCF at the Rh-HK’s surface followed a modified first-order kinetics, most probably by catalytic hydrodehalogenation and subsequent hydrogenation of the aromatic moieties (molecular hydrogen was provided by the corrosion of Mg). From the determined reaction rate constants at four different temperatures, the activation energy was estimated to be 59.6 kJ/mol by means of the Arrhenius equation what is in good agreement with similar results reported in the literature. This coupled hydrodehalogenation and hydrogenation approach may be upscaled into a new promising technical process for comprehensively removing such pharmaceuticals and similar pollutants in sewage plants in a single step, furthermore, even in combination with adsorption by activated carbon and/or ozonation which have already been established at some sewage plants in Switzerland and Germany recently.

**Supplementary Information:**

The online version contains supplementary material available at 10.1007/s11356-024-32777-1.

## Introduction

More than 3,000 active pharmaceutical ingredients are administered worldwide in prescription, over-the-counter, and veterinary medicines in addition (aus der Beek et al. 2016). Its active ingredients include various synthetic compounds manufactured by pharmaceutical companies worldwide in quantities of 100,000 tons per year (Weber et al. 2014). The increasing and ageing of the global population as well as advances in research, development, and medical care have led to a significant increase in drug use in the last two decades (Van Boeckel et al. 2014). The medicines taken are often only incompletely metabolized by the human body. Therefore, drugs and their transformation products enter the municipal sewage systems via human waste expulsion (Babu et al. 2009; Awfa et al. 2018). Medicines improperly disposed of and drained also end up in the sewage system with identification of more than 100 different pharmaceutical substances (aus der Beek et al. 2016). Furthermore, inputs from agriculture, industrial production, medical facilities, aquaculture, and the spreading of contaminated liquid manure as fertilizer are of local relevance (Weber et al. 2014).

The occurrence of pharmaceutical residues is a huge problem worldwide, and around 560 different pharmaceuticals were detected in 2016 (aus der Beek et al. 2016). In various research, wastewater samples, inclusive of both inflows and outflows from sewage treatment facilities, along with sewage sludge, were systematically collected for analysis. It is plausible to infer a correlation between the presence of pharmaceutical residues in wastewater treatment plants and surface water bodies, such as river or lakes. This association arises from the direct discharge of the effluents from wastewater treatment plants, thereby necessitating an in-depth exploration of the interlinkages between pharmaceutical contaminants in wastewater and their impact on surrounding aquatic ecosystem (Baker and Kasprzyk-Hordern 2013; Weber et al. 2014; Sharma et al. 2019). Most of the pharmaceutical substances are often persistent compounds that are difficult to biodegrade. In many cases, conventional municipal sewage treatment plants cannot break down the persistent pharmaceutical residues with sufficient efficiency within the framework of their biological purification stages consisting of nitrification and denitrification (Mirzaei et al. 2018). Nevertheless, retention of pharmaceutical residues in sewage treatment plants can be observed, which can be attributed to partial degradation in the biological purification stage and to adsorption on sewage sludge (Xiang et al. 2018). However, the partial biodegradation of pharmaceutical residues often does not lead to a desired complete mineralization of the pollutants but to the formation of transformation products with unknown properties (Mirzaei et al. 2018; Morales-Paredes et al. 2022). Long reaction times, which can be on the order of hours to months, are often disadvantageous (Baena-Nogueras et al. 2017; Schmidt et al. 2017). Therefore, further treatment processes are required to accelerate the breakdown of pollutants in order to demineralize a wide range of substances.

Over the years, various other treatment methods, besides biodegradation, have been adopted in various research works to treat different pharmaceutical contaminants. Among them, ultraviolet (UV) photodegradation (Baena-Nogueras et al. 2017), advanced oxidation processes chiefly covering Fenton’s reagents technique, ozonation techniques, and heterogeneous photocatalytic approaches (Mohapatra et al. 2014; Bian et al. 2014; He et al. 2016, 2021; Gomes et al. 2017; Almomani et al. 2018; Di et al. 2019; Wang and Chen 2020; Bisaria et al. 2021; Issaka et al. 2022), electrochemical oxidation processes (Babu et al. 2009), and sorptive processes (Kårelid et al. 2017) are some of the significant methods which were investigated regarding upscaling to technical applications of sewage treatment. However, these methods exhibited certain drawbacks in terms of the involved attenuation/degradation potentials, primarily attributed to challenges related to the interference of inorganic ions, heightened toxicity resulting from transformed products, the impact of possible water turbidity on the degradation process and/or complications associated with fouling processes (Schulze et al. 2010; Tijani et al. 2014). Therefore, catalytic dehydrodehalogenation or hydrogenation emerged as one of the promising alternative methods to provide maybe more efficient degradation solutions for various pollutants in aqueous solutions (De Corte et al. 2012; Chaplin et al. 2012; Mackenzie et al. 2006; Patel and Suresh 2007; Miyamura et al. 2018).

Catalytically active metals from the group of transition elements/metals such as Cu, Ni, Pd, platinum (Pt), and Rh are able to convert molecular hydrogen into ad- or even absorbed highly reactive atomic hydrogen serving as a strongly reducing agent (Stanislaus and Cooper 1994; Mackenzie et al. 2006; Patel and Suresh 2007; Gao et al. 2016; Miyamura et al. 2018; Rayhan et al. 2018). Such activated hydrogen can be added to double and triple bonds of organic molecules and also reduce certain functional groups such as halogen atoms attached to saturated or unsaturated C-C bonds (such as vinyl or aromatic moieties of a certain molecule). That is, hydrodehalogenation reactions are also possible (Mackenzie et al. 2006; Zhu and Lim 2007). With Pd as a catalyst, organochlorine compounds such as 4-chlorophenol as well as chloroalkanes and chloroalkenes can be reductively dechlorinated (Baeza et al. 2012; Chaplin et al. 2012). In other words, a more selective reaction process for the degradation of pharmaceuticals, personal care products and other emerging pollutants may stand for an advantageous of application of catalytic hydrogenation in green/sustainable chemistry. This might be in contrast to oxidative processes, which can produce a wide range of intermediates and final reaction products and by-products, some of which have unknown toxicological behavior (de Gusseme et al. 2012). In comparison to Pd, with the choice of another suitable catalyst such as Rh, the hydrogenation of aromatic systems may also be possible or even more effective (Alsalahi et al. 2018).

Since the 1990s, zero valent metals, especially ZVI but also ZVM have emerged as a promising approach/reagents to eliminating different pollutants in groundwater and wastewater predominantly by reductive reactions, for instance, in permeable reactive barriers (PRBs) regarding ZVI (Li et al. 2008; Fu et al. 2014, Birke et al. 2014; Singh et al. 2023). Zero-valent metals have proven to be suitable for reducing pollutant concentrations in the case of chlorinated organic compounds (Fu et al. 2014). However, the degradation of various pharmaceuticals proves to be limited when utilizing only zero-valent metals as the sole reducing agent due to rapid passivation of reactive sites, low removal efficacy etc. (Dong et al. 2018). Therefore, doping or applying mixtures of zero-valent metals along with different catalytic metals such as Cu, Ni, Pd, Pt, and Rh proves as a potentially suitable approach for enhancing the zero-valent metal's reactivity and therefore reducing the actual concentration of various parent recalcitrant pharmaceutical substances in aqueous solutions (Ghauch et al. 2010, 2011; Gao et al. 2016). The mechanism of pollutant elimination using doping metals is based on the electrochemical corrosion of the zero-valent metals, which supplies electrons ($M{g}^{0}\to M{g}^{2+}+2{e}^{-}; F{e}^{0}\to F{e}^{2+}+2{e}^{-}$) to form reactive species such as OH radicals and/or adsorb or absorb molecular hydrogen to generate activated (atomic) hydrogen for dehydrohalogenation and/or hydrogenation (Harada et al. 2016; Yamaguchi et al. 2018; Gao et al. 2016). Fe-Pd bimetal was effective in dechlorination (Fu et al. 2014), however, Pd was identified as the active site on the Fe-Pd surface for various dechlorination reactions and proven very effective for diclofenac (DCF) hydrodehalogenation (Zhu and Lim 2007). Similarly, Fe-Cu and Fe-Ni bimetal also proved suitable for degradation of different pharmaceutical substances (Ghauch et al. 2010). Similarly, Mg-Pd was also utilized as an innovative and efficient bimetal alternative for hydrodechlorination of pentachlorophenol, 2,4,5-trichlorophenol and 2-chlorophenol (Patel and Suresh 2007). Mg-Pd may produce activated hydrogen in aqueous solution during corrosion once initiated with small portions of ammonium chloride added. Catalytic hydrodechlorination and/or hydrogenation of aromatic moieties may subsequently facilitate the degradation of a variety of pharmaceutical substances significantly, because a larger number of them comprise one or more aromatic rings, some of them substituted by halogen atoms (DCF, iomeprol etc.). However, a detailed investigation of a variety of pharmaceutical contaminants degradation with different bimetal reagents following the catalytic hydrogenation pathway of aromatic moieties is still required.

Therefore, the primary objective of this study was to investigate the degradation of DCF, IBP, EE2 and BPA in aqueous solutions applying catalytic hydrogenation conditions by using Mg-HKs, however, comparing them with some ZVI bimetalls which were already investigated previously (Ghauch et al. 2010, 2011). Therefore, micro-iron particles, ZVI bimetals, as well as ZVM, and Mg-HK combinations were employed. Moreover, this study also investigated the heterogeneous degradation kinetics involved in the catalytic dehydrohalogenation and hydrogenation of DCF in water using a ZVM-Rh-HK combination by successfully fitting a suitable non-linear kinetic model to various experimental data.

## Materials and methodology

### Chemical reagents and reactive materials

Deionized water was used for sample preparations showing a conductivity of less than 20 µS/cm. The chemicals utilized during the experiments were procured from various manufacturers (Table S1 (supplementary information)). Iron powders were stored in amber glass bottles under an argon (Ar) atmosphere to prevent atmospheric oxygen reactions. Specifically, Würth iron powder (GH-K) remained in the clip bags, and following each iron material extraction, the bag contents were purged with Ar to minimize excess gas volume. A sealed pouch was then placed into the amber glass bottle, purged with Ar, and sealed with parafilm to maintain an inert environment. Same procedure was followed for Mg powder in the storage container, ensuring it remained under an Ar atmosphere, with the closure thread also sealed using parafilm.

### Experimental setup

Various short-term batch experiments were conducted for analyzing the attenuation (degradation) potentials of ZVI, ZVI bimetals, ZVM, and ZVM combinations with HK (Rh, Pd, Ru on alumina supports for three different pharmaceutical contaminants as model pollutants, i.e., DCF, IBP, EE2, and BPA (as another emerging pollutant that is widely employed in the manufacturing of various plastics such as linings of cans)). The detailed methodology of all the experiments is explained below in the further subsections. All ZVI and ZVI bimetal experiments were conducted on an overhead shaker within a climate chamber and all ZVM and ZVM bimetal experiments were conducted on magnetic stirrers equipped with a heating plate and heating bath to ensure a constant temperature. This was supplemented by heatable CMAG HS 7 magnetic stirrers and digital contact thermometers from IKA Werke GmbH & Co. KG, Germany, and an overhead shaker (Gesellschaft für Labortechnik, GFL 3040, Germany). Dissolved oxygen measurements were made with a CellOx 325 probe (Xylem Analytics GmbH, Germany).

### Batch experiments employing iron bimetals

Numerous experiments were conducted with iron bimetals, wherein non-ferrous metals were doped onto the iron surface (O’Carroll et al. 2013). ZVI powder was supplied by "Eisenwerke Würth", Bad Friedrichshall, Germany ("Würth"). Considering the standard hydrogen electrode, Cu, and Ni exhibit higher redox potentials rather than the Fe/Fe^2+^ half cell (Table S2 (supplementary information)). Therefore, in this investigation, Cu and Ni were doped on the iron’s surface using a water-based method. A small amount of water-soluble salts containing the respective metal was weighed concurrently with ZVI. With the addition of the sample solution, the salt dissolves, initiating a reduction and a deposition of the doping metal on the ZVI surface. For divalent metal (Me) ions, the deposition process can be described by the following equation.

1 $$M{e}^{2+}+Fe\to Me+F{e}^{2+}$$

The sample solutions, outlined in Table 1, were freshly prepared daily. Experiments comprising DCF were conducted in triplicate batch experiments, employing two different iron mass concentrations (*C*_Fe_), 20 g (*C*_Fe_ = 200 g/L) or 40 g iron (*C*_Fe_ = 400 g/L), weighed into 300 mL Erlenmeyer flasks. Salts of the doping metals (Cu and Ni with C_b_ = 250 μmol salts/g Fe each) were weighed in parallel to the iron. Attenuation tests for sample substance mixtures were also conducted at a mass concentration of *C*_Fe_ = 50 g/L in 100 mL Erlenmeyer flasks. The initial mixing concentration (*C*_mix0_) for each sample substance was 100 µg/L as shown in Table 1.

**Table 1:** Model pollutants and associated concentrations in the attenuation tests using ZVI (Würth Eisenwerke, Germany)

Sample substance	Molecular weight in g/mol	Single substances		Pollutant mixture	
		*C*_0,__pollutant_ in mg/L	*C*_0,__pollutant_ in µmol/L	*C*_mix0,_ _pollutant_ in µg/L	*C*_mix0,_ _pollutant_ in µmol/L
DCF (sodium salt)	318.10	10	31.43	100	0.314
IBP (sodium salt)	228.26	10	43.81	100	0.438
BPA	228.29	10	43.80	100	0.438
EE2	296.40	10	33.74	100	0.337

The experiments were conducted at 25 °C temperature. The samples were mixed at 5 revolutions per minute (rpm) in an overhead shaker. During sampling, a 1 mL sample was taken and filtered through a 0.45 µm syringe filterpolytetrafluoroethylene (PTFE) filter. Slowly pushing the samples through the filter was observed to be advantageous for better retention of fine particles. Initial tests with iron bimetals revealed the necessity of sample processing due to precipitation of iron compounds (iron hydroxides / oxides) in the sample vial, potentially leading to instrumental blockage. Consequently, after sampling, 500 µL of the filtered sample was diluted with 500 µL of diluted hydrochloric acid (0.01 mol/L).

### Batch experiments in the presence of buffer

Experiments also aimed at (semi-quantitatively) identifying adsorption processes, which might have occurred in parallel with actual degradation reactions. They were performed applying a slightly modified experimental setup. Weighing was executed in 300 mL Erlenmeyer flasks, with the addition of 100 mL of the respective sample solution, aligning with concentrations specified in Table 2. Following a 45 min interval, 100 mL of acetic acid-acetate buffer (2 mol/L) was added, and the samples were further mixed in an overhead shaker at 5 rpm.

**Table 2:** Degradation of individual substances with iron bimetals when buffering after 45 min for analyzing the adsorption effect during the degradation process (*C*_Fe_ = 50 g/L; *C*_*b*_ = 250 µmol/g Fe; *ϑ* = 25 °C)

Sample substance	*C*_0_ in µmol/L	*C*_*t*_ until 45 min in µmol/L		*C*_*t*_ after addition of buffer in µmol/L		Recovery related to *C*_0_ in %	
	Both bimetals	Fe-Cu	Fe-Ni	Fe-Cu	Fe-Ni	Fe-Cu	Fe-Ni
DCF	31.43	0.69	2.94	15.38	10.84	46.7	25.1
IBP	43.81	24.51	35.01	26.30	37.84	4.1	6.5
BPA	43.80	35.26	40.05	32.02	36.95	− 7.4	− 7.1
EE2	33.74	6.83	27.56	8.09	27.54	3.7	0

### Batch experiments comprising catalytic hydrogenation

The test solutions were freshly prepared each day. Different masses of Mg powder (5 g, 10 g, 15 g, and 40 g) were precisely weighed into 300 mL Erlenmeyer flasks for the degradation tests. Subsequently, 100 mL of DCF solution (10 mg/L or 31.43 µmol/L) was added, and mixing was carried out with a magnetic stirrer at 1,000 rpm. All experimental procedure were conducted at 25 °C temperature inside a climate chamber.

Degradation tests were performed in a modified test setup with Mg powder (2.5 g) and hydrogenation catalysts, i.e., Pd-HK, Rh-HK, or Ru-HK, which had been applied to an aluminum oxide (Al_2_O_3_) support each with a mass fraction of 5 % for each catalyst metals (purchased from suppliers). For hydrogenation degradation experiments, 2.5 g of Mg powder and the respective amounts of the hydrogenation catalyst were weighed into 300 mL Erlenmeyer flasks. 100 mL of the respective pollutant solution was poured into 300 mL Erlenmeyer flasks. The reaction was initiated by introducing 1 mL of ammonium chloride solution (0.5 mol/L), thus, promoting the corrosion of Mg, that is, producing molecular hydrogen and hydroxide. Mixing was performed with a magnetic stirrer at 1,000 rpm, and temperature control was achieved using a climatic chamber and water baths.

### Analytical method

The analysis of all samples was conducted using an LC-MS-MS system consisting of a Waters 2695 HPLC additionally equipped with a Waters 2487 UV/Vis detector. The instrumental parameters for the methods are detailed shown in Table S3 (supplementary information). A Waters Micromass Quattro LC served as the quadrupole MS, and its method parameters can be found in Table S4 (supplementary information). The chromatographic setup included a C18 reversed-phase column (Reprosil-Pur 120 C18-AQ, 50 mm × 2 mm, 3 µm) with a pre-column cartridge (10 mm × 2 mm) from Dr. Maisch, along with a ZORBAX Eclipse Plus C18 (250 mm × 4.6 mm, 5 µm) from Agilent. For a single substance analysis, the UV/Vis detector method (ME2 which is modified from method ME1) was used with the measuring range spanned from 190 to 700 nm, with DCF, IBP, BPA, and EE2 detected at wavelengths of 275, 220, 195, and 200 nm, respectively. Method EE3 was developed using the MS detector to separate the sample substance mixture and analyzing the byproducts of parent pollutants (focussing on DCF, BPA). The separating column was set up in an adjustable column oven, and gradient methods were employed, altering the eluent composition over the runtime. Water and acetonitrile (ACN), with the addition of 0.1 % formic acid, were used as eluents.

## Results and discussion

### Degradation with iron bimetals

**Fig. 1:**
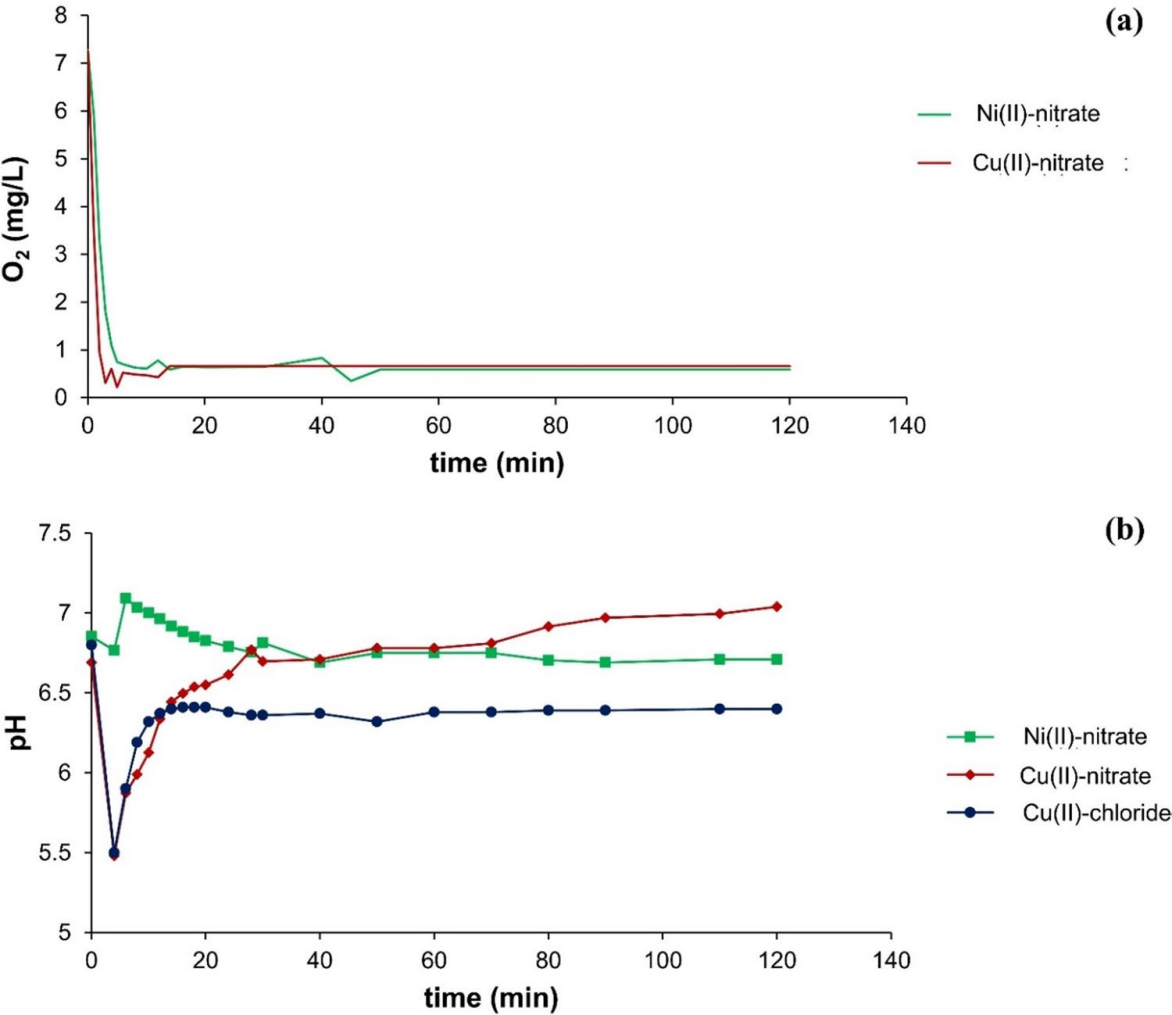
**a** Dissolved oxygen when adding Fe-Cu or Fe-Ni bimetal at 25 °C (*C*_Fe_ = 50 g/L; doping salt (*C*_b_) = 250 µmol/g Fe), **b** pH values at 25 °C when adding Fe bimetals with different doping salts, i.e., Ni(II)-nitrate, Cu(II)-nitrate, and Cu(II)-chloride (*C*_Fe_ = 50 g/L; *C*_b_ = 250 µmol/g Fe)

When the Fe-Cu or Fe-Ni bimetal was added to water, a decrease in dissolved oxygen could be observed. Initially, the dissolved oxygen concentration was measured at 7.2 mg/L at a temperature (*ϑ*) of 25 °C. Within the first 10 min, both metal combinations exhibited a decrease, reaching around 0.6 mg/L, and remained nearly constant over a 120 min period (Fig. 1a). The initial pH of deionized water was approximately 6.7. With the addition of Fe-Ni bimetal, the pH briefly rose to 7.1 before reverting to the initial value of 6.7. Conversely, when introducing Fe-Cu bimetal, the initial pH drop to 5.5, succeeded by a subsequent increase to pH 7. Similarly with Fe-Cu bimetal, the pH value also dropped to 5.5, followed by an increase to a value of 6.4 (Fig. 1b).

**Fig. 2:**
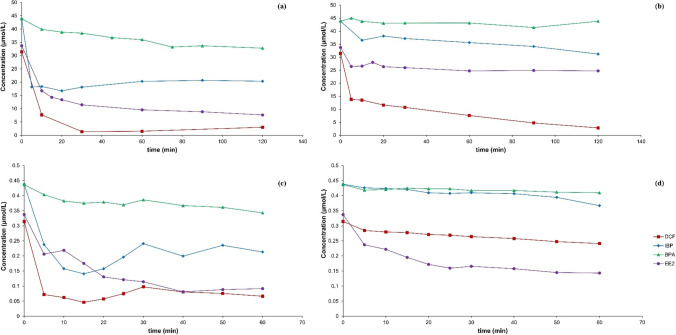
Attenuation at 25 °C of **a** a single pollutant substance (DCF, IBP, EE2, and BPA in separate solutions each) with Fe-Cu bimetal (*C*_Fe_ = 50 g/L; *C*_b_ = 250 µmol/g Fe; *C*_0,__pollutant_ = 10 mg/L), **b** a Single pollutant substance with Fe-Ni bimetal (*C*_Fe_ = 50 g/L; *C*_b_ = 250 µmol/g Fe; *C*_0,__pollutant_ = 10 mg/L), **c** mixtures of DCF, IBP, EE2, and BPA in one solution with Fe-Cu bimetal (*C*_Fe_ = 50 g/L; *C*_b_ = 250 µmol/g Fe; *C*_mix0__, pollutant_ = 100 µg/L), **d** Mixtures in one solution with Fe-Ni bimetal (*C*_Fe_ = 50 g/L; *C*_b_ = 250 µmol/g Fe; *C*_mix0__, pollutant_ = 100 µg/L)

The initial concentrations of the single substance, for individual substance and mixture substances solutions, were 100 µg/L for each sample substance. The attenuation of single substances, covering DCF, IBP, EE2, and BPA each, are shown in Fig. 2a–b and c–d, respectively. Among all the single substance solutions, DCF showed better attenuation rates with Fe-Cu (Fig. 2a) and Fe-Ni (Fig. 2b) bimetals compared to other single substances. In both cases, DCF was attenuated at more than 90 %; however, the attenuation rates for other substances, i.e., IBP, EE2, and BPA, were slower and more efficient with Fe-Cu compared to Fe-Ni.

Contrarily, the attenuation rates of mixed substances within a single solution showed variations with iron bimetals when compared to individual substance solutions of each contaminant. For Fe-Cu bimetal, the results showed, after 1 h (Fig. 2c), an attenuation of DCF, IBP, EE2, and BPA at about 79 %, 51 %, 73 %, and 21 %, respectively. For Fe-Ni bimetal, after 1 h (Fig. 2d), decreases in contaminant concentrations to 23 %, 16 %, 58 %, and 6 % for DCF, IBP, EE2, and BPA were observed, respectively.

In conclusion, ZVI is able to attenuate a number of organic compounds in the presence of dissolved oxygen. "Attenuation" may comprise actual degradation and adsorption processes, however, no degradation products could be identified by LC-MS analyses. Actual pollutant degradation may be based on the electrochemical corrosion of iron associated with an electron transfer from iron that may form hydrogen peroxide (H_2_O_2_) in the presence of dissolved oxygen (Eq. 2) (Fu et al. 2014). The formation of H_2_O_2_ leads to a decrease in the concentration of dissolved oxygen (Harada et al. 2016).

2 $$Fe+{O}_{2}+2{H}^{+}\to F{e}^{2+}+{H}_{2}{O}_{2}$$

The formation of H_2_O_2_ can serve as a starting material for the formation of hydroxyl radicals (Eq. 3), which have a high oxidation potential compared to organic compounds (Fu et al. 2014). This is described as a Fenton-like response (Harada et al. 2016). The hydroxide ions give rise to an increase in the pH value.

3 $$F{e}^{2+}+{H}_{2}{O}_{2}\to F{e}^{3+}+\cdot OH+O{H}^{-}$$

Previous tests have shown that only a small reduction in the DCF concentration can be achieved with undoped iron. By depositing more noble non-ferrous metals, it is possible to increase iron reactivity. Iron serves as a base metal (*E*^0^ = −0.44 V), that is, as a reducing agent. The deposition of more noble metals such as Cu or Ni on the surface of the iron particle leads to the formation of an iron-nonferrous metal local element. The formation of the local element accelerates the corrosion of iron, which is relevant for the breakdown of pollutants (O’Carroll et al. 2013). In addition, dehalogenation reactions on the iron's surface are possible (Li et al. 2008). As iron corrosion advances, it results in the formation of an amorphous layer of corrosion products, primarily composed of iron oxides and/or iron hydroxide and/or iron oxide hydroxide forming on the surface of the iron particle (Ghauch et al. 2010; Yan et al. 2010). This manifest visually as a red-brownish discoloration of both the iron material and sample solution. Those layers serve as an adsorbent for pollutants such as DCF (Ghauch et al. 2010), IBP (Yin et al. 2018), and their degradation products (Fujioka et al. 2016). The core of this structure comprises ZVI, which has reductive properties for the breakdown of pollutants; therefore, an iron particle can be described by a core-shell model (Li et al. 2008).

DCF should firstly be adsorbed onto the ZVI's surface and iron corrosion products deposited on the ZVI's surface while its corrosion in water. As a result of the morphological changes on the particle surfaces due to the progressive iron corrosion, the already adsorbed DCF should be released into the aqueous solution again (Ghauch et al. 2010). The speed of the deposition of the non-ferrous metals on the iron surface depends on the standard potential *E*^0^ of the redox couple of the metals to be deposited. The normal potential of the Cu/Cu^2+^ half cell at +0.34 V is significantly may positive than that of the Fe/Fe^2+^ half cell at −0.44 V, which results in a rapid reduction of the Cu^2+^ ions. However, the redox potential of the Ni/Ni^2+^ half cell at −0.28 V is only slightly more positive than that of Fe/Fe^2+^, and sorption and reduction may take place more slowly (Ling et al. 2017). The more rapid deposition and thus more rapid formation of the contact corrosion element explain the accelerated decrease in the pollutant concentration in the Fe-Cu bimetal compared to the Fe-Ni bimetal. The brown discoloration of the sample solution due to iron corrosion products occurred more slowly with the Fe-Ni bimetal than with the Fe-Cu bimetal. The deposition of zero-valent Cu on the surface increases adsorption and reductive degradation capacity without stimulating the formation of OH radicals or oxidative reactivity. Cu accelerates the passivation of the iron surface and inhibits the formation of OH radicals (Harada et al. 2016; Yamaguchi et al. 2018).

What is striking is the fact that the decrease in concentration in the DCF degradation tests differs depending on whether Cu(II)-nitrate or Cu(II)-chloride is used as the doping salt. The redox potential of the redox couple NO_3_^−^-NH_4_^+^ is +0.884 V. ZVI is able to reduce nitrate (Fu et al. 2014). A nitrate ion can be reduced with the formation of iron corrosion products such as Fe_3_O_4_ (Eq. 4) (Suzuki et al. 2012).

4 $$N{O}_{3}^{-}+3Fe+{H}_{2}O+2{H}^{+}\to N{H}_{4}^{+}+F{e}_{3}{O}_{4}$$

The brown discoloration develops optically faster when using Cu(II)-nitrate rather than when using Cu(II)-chloride. The additionally formed iron corrosion products represent an adsorbent for the pollutants and their degradation products (Ghauch et al. 2010; Yin et al. 2018). The presence of nitrate ions accelerates iron corrosion using a Fe-Cu bimetal, and it could have an inhibiting effect on the degradation of pollutants (Dong et al. 2018). This is due to the fact that accelerated iron corrosion leads to the rapid formation of the oxide shell on the iron particles. The iron core is passivated so that the adsorption increases, and the pollutants’ breakdown is inhibited. This becomes clear using the example of DCF degradation by means of Fe-Cu bimetal using Cu(II)-nitrate as a doping salt. The rapid formation of the oxide shell leads to a large adsorption capacity so that the DCF concentration decreases in a short time. The experimental findings underscore the potential of Fe-Cu and Fe-Ni bimetals in efficiently attenuating organic pollutants in water systems, with DCF exhibiting particularly high degradation and/or adsorption (attenuation) rates. The deposition of noble non-ferrous metals on iron particles may enhance rates of adsorption and/or degradation. However, the choice of doping salt influences the attenuation process, with Cu(II)-nitrate accelerating iron corrosion but potentially inhibiting pollutant breakdown due to passivation of the iron core. Overall, the observed attenuation processes appear to take place due to combined mechanisms of adsorption and degradation, emphasizing the complexity of pollutant removal with these bimetals and highlighting the need for further investigation to optimize water treatment strategies using ZVI bimetals.

### Adsorption analysis by adding buffer

**Fig. 3:**
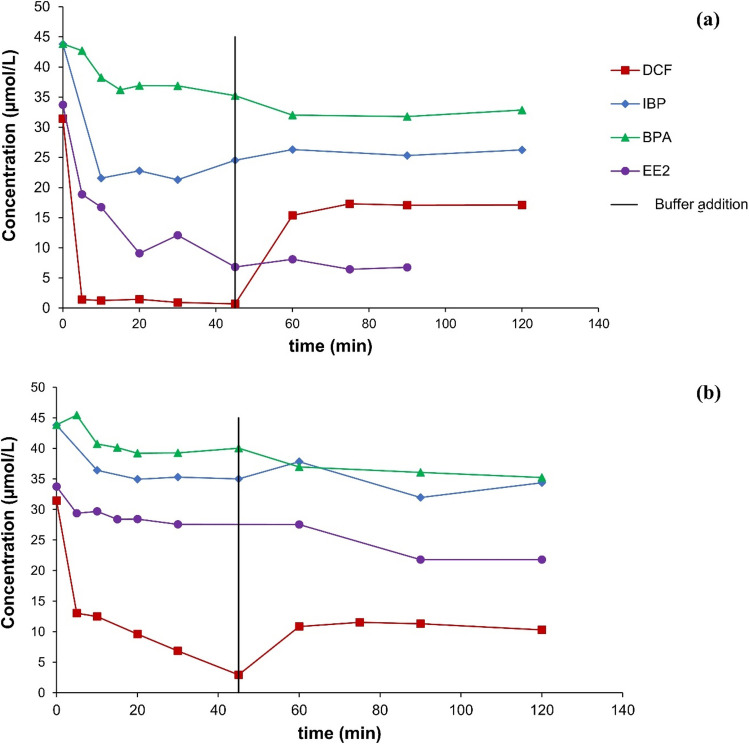
Concentration curves involving buffering after 45 min at 25 °C **a** with Fe-Cu bimetal (*C*_Fe_ = 50 g/L; *C*_b_ = 250 µmol/g Fe; *C*_0,__pollutant_ = 10 mg/L) and **b** with Fe-Ni bimetal (*C*_Fe_ = 50 g/L; *C*_b_ = 250 µmol/g Fe; *C*_0,__pollutant_ = 10 mg/L)

In the buffered experiments, after an experiment run time of 45 min, an increase of the DCF concentration could be observed. In the case of Fe-Cu bimetal, a DCF release from 0 to 55 % of its initial concentration could be determined (Fig. 3a). When using Fe-Ni bimetal, the concentration of the DCF increased from 9 to 37 % of the initial concentration (Fig. 3b). For BPA and EE2, the concentration curves, after buffering the test mixture, showed no increase in the concentration again. For IBP, slight increases of about 5 % of the initial concentration were recorded for both bimetals (Table 2). Buffering with acetic acid-acetate buffer to a pH value of 4.75 partly dissolves the oxide layer, as its formation depends on the pH value (Fujioka et al. 2016). This shows that regarding Fe-Cu bimetal, adsorption of DCF on iron corrosion products should be largely held responsible for the decrease in the DCF concentration in the sample solution, probably at least 55 % or even more. A quantitative release of degradation products is not to be expected in the case of DCF due to the strongly adsorptive effect of the metal corrosion products (Ghauch et al. 2010). This approach (that is, adding an acidic buffer after a certain reaction time) highlights the importance of understanding the interplay between pH, adsorption, and degradation mechanisms in optimizing pollutants removal using bimetal systems and may be useful in future examinations, too.

### Degradation employing Mg

The concentration curves (Fig. S1 (supplementary information)) showed a decrease in the DCF concentration when using Mg powder. The greatest decrease in concentration was observed at a mass concentration of 400 g/L. Within 10 min, the concentration drops to 36 % of the initial concentration; after 120 min, 27 % of the initial DCF concentration remains. At mass concentrations of 150, 100, and 50 g/L, residual concentrations of 41 %, 52 %, and 69 % of the initial concentration were found after 120 min. Hence, the use of Mg powder showed a decrease in the DCF concentration which increased as the mass concentration of Mg increases. In water, Mg forms a poorly soluble coating of magnesium hydroxide (Mg(OH)_2_) as well as a finely dispersed precipitate (Eq. 5) with the evolution of hydrogen, which passivates the Mg particles and/or may serve as an adsorbent/flocculant, respectively.

5 $$Mg+2{H}_{2}O\to {H}_{2}+Mg{(OH)}_{2}$$

Apart from the DCF peak, the chromatograms did not show any other peaks that indicated formed breakdown products. The initially clear and then stagnant decreases in the DCF concentration indicated adsorption processes. In relation to the measured attenuation of the concentration of DCF, comparatively high Mg mass concentrations were required. The use of Mg as a reactive metal requires activation to counteract surface passivation and promote hydrogen evolution and activation. This was conducted in combination with a catalyst. Remarkably, at Rh-HK mass concentrations of 1,000 and 2,500 mg/L, complete decreases in DCF concentrations were observed after 60 and 30 min, respectively. If 10 mg/L Rh-HK was used per batch, DCF was not completely removed after 60 min and 57 % of the initial concentration remained in the solution. Note that without using the Rh catalyst, only a slight decrease in the DCF concentration to 92 % of the initial concentration could be observed. If equimolar amounts of Cu, Ni, or Ru-HK were used, only slight decreases in the DCF concentration of up to 20 % could be found (Fig. S2a (supplementary information)). Similarly, Fig. S2b (supplementary information) shows IBP concentration degradation curves. At Rh-HK mass concentrations of 1,000 and 2,500 mg/L, decreases in IBP concentrations to 6.0 and 0.5 mg/L, respectively, were observed after 120 min. Due to these findings, investigations were focused on Mg-HK to treat the model pollutants.

### Degradation employing Mg and Rh-HK

**Fig. 4:**
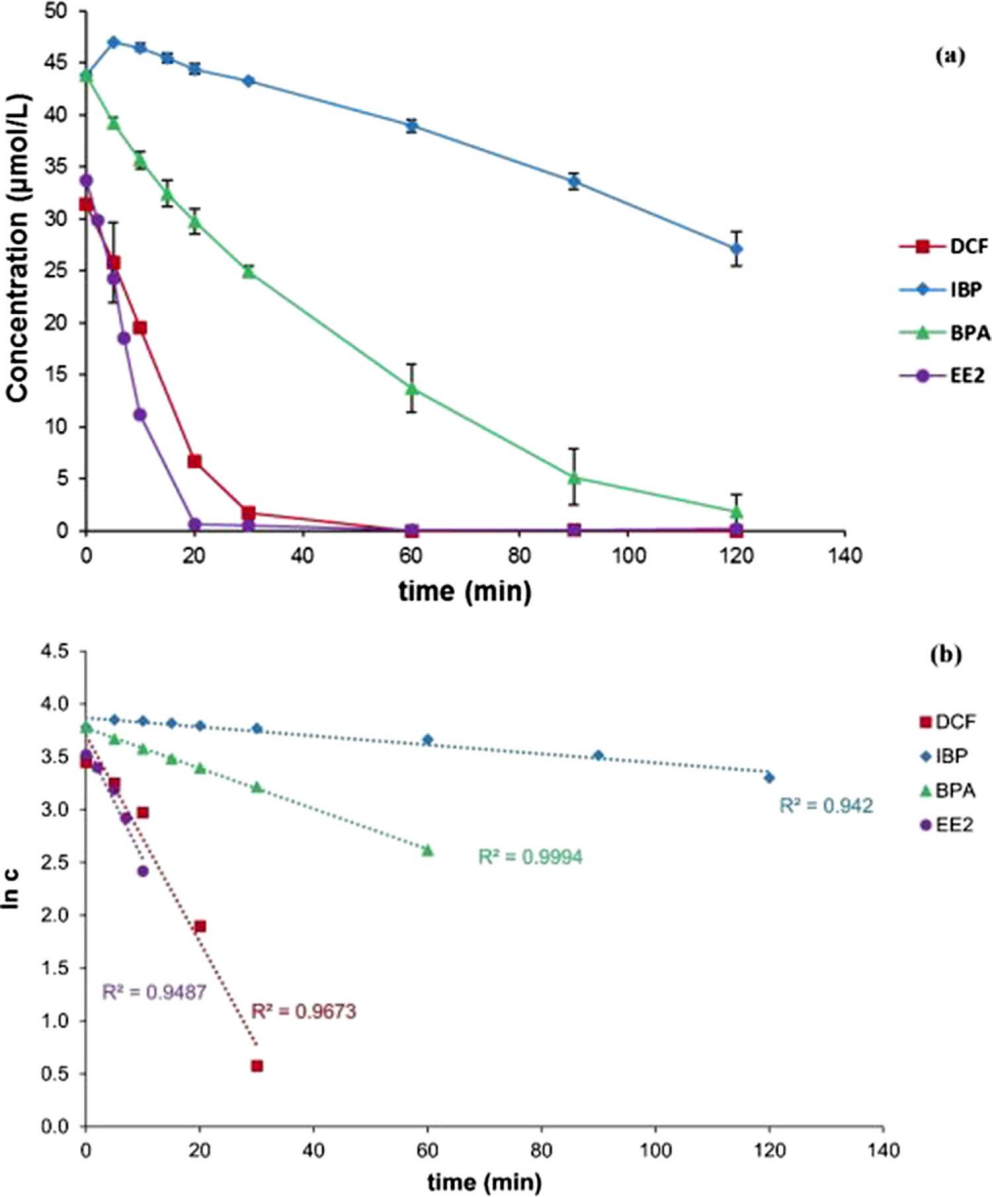
**a** Sample substance concentration curves for application of Mg and Rh-HK to DCF, IBP, EE2, and BPA in aqueous solution each at 25 °C (*C*_Mg_ = 25 g/L; *C*_Rh-HK_ = 1,000 mg/L; *C*_0,__pollutant_ = 10 mg/L); **b** Plot of ln c versus time (t)

Figure 4a reveals the concentration curves of individual sample substances at a mass concentration of Rh-HK of 1,000 mg/L employed each. Decreases in concentrations were also observed for EE2 and BPA. Remarkably, EE2 was completely removed after 20 min. BPA was almost completely removed after 2 h. The degradation of all four substances showed a good correlation with a pseudo first-order degradation kinetics relationship, as shown in Fig. 4b for the plot of ln c of each substance against time. The trend also follows the same published in a previous publication, although covering photolysis and biodegration, but not hydrogenation (Baena-Nogueras et al. 2017). In addition, these findings indicated that actual chemical degradation was most probably responsible for the decrease in the model pollutant’s concentrations.

Activating the Mg with ammonium chloride and using Rh-HK showed an accelerated decrease in the pollutant concentration. In comparison to the non-catalyzed experiments, lower magnesium weights of 2.5 g per test batch were required. Note that, ammonium chloride serves as a weak acid and therefore dissolves protective hydroxide layers at the surface of Mg (Eq. 6), hence, Mg may readily and completely corrode in water by producing molecular hydrogen and hydroxide which, however, no longer protects the metal from dissolution in water.

6 $$Mg{(OH)}_{2}+2N{H}_{4}Cl\to MgC{l}_{2}+2N{H}_{3}+2{H}_{2}O$$

In other words, this counteracts the passivation of the magnesium surface to be visually seen by the evolution of gas on the surface of the magnesium particles dispersed in the aqueous solution in the flask. After the start of the experiment, the pH value rose to a value between 10 and 11, which was caused by the formation of hydroxide ions. The added catalyst, however, is responsible for the accelerated decrease in pollutant concentrations. Rh has high catalytic activity for catalytic hydrodechlorination and hydrogenation (Lokteva et al. 1989; Alsalahi et al. 2018). The catalyst is required for the cleavage of the relatively stable H–H bond in the hydrogen molecule, leaving metal-bonded hydrogen atoms on the catalyst’s surface. This activated hydrogen readily reacts with multiple bonds of co-adsorbed pollutant molecules. In addition, effective hydrodechlorination reactions at the catalyst’s surface are also possible. The rapid decrease in the DCF concentration can be explained by two initial (stepwise) hydrodechlorination reactions (due to the analyses of the degradation products by LC-MS-MS, see below), in which activated hydrogen eventually converts in a well-known mechanistic step the two C-Cl bonds of the aromatic ring to C-H bonds each (Mackenzie et al. 2006).

**Fig. 5:**
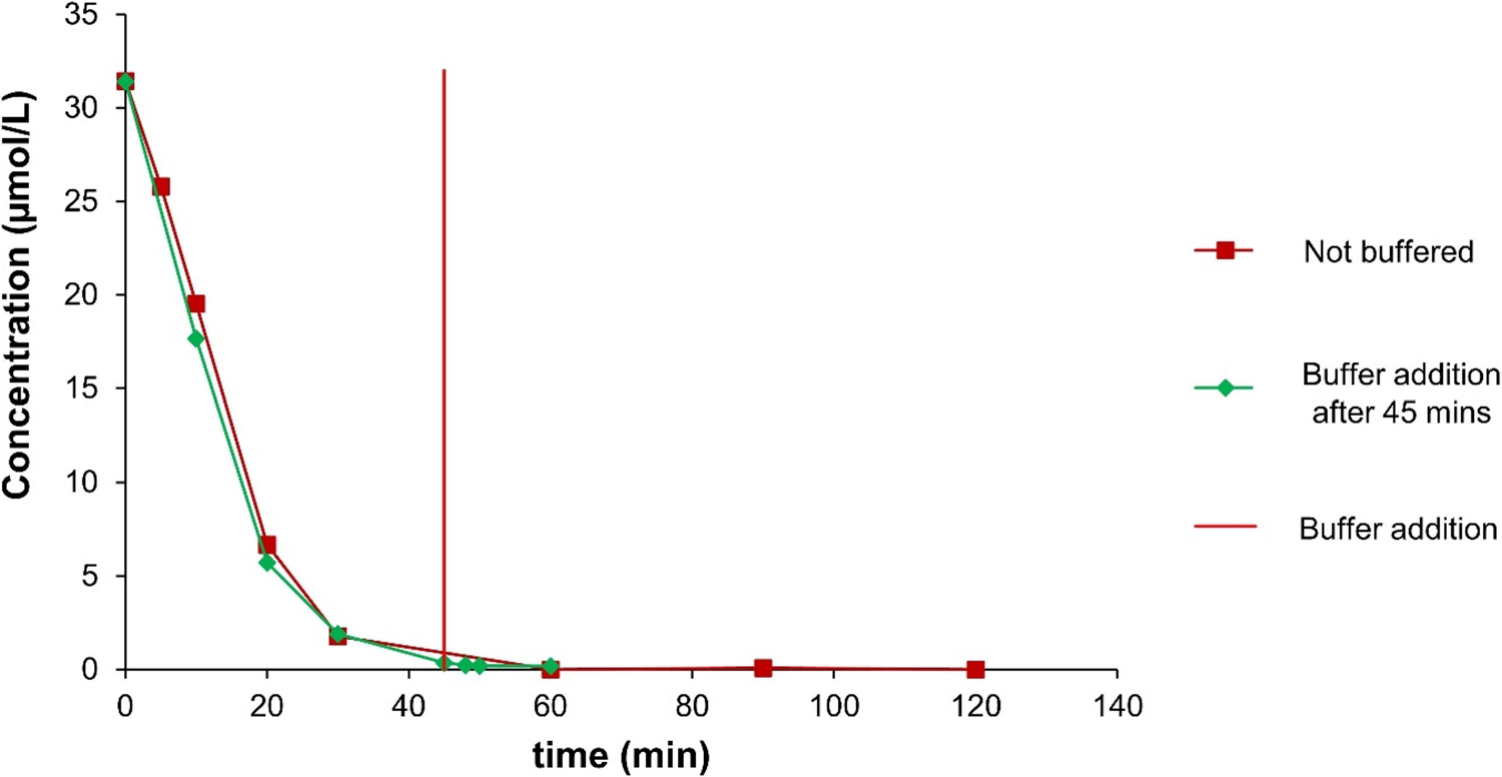
DCF concentration course with Mg and Rh-HK with buffering after 45 min at 25 °C (*C*_Mg_ = 25 g/L; *C*_Rh-HK_ = 1,000 mg/L; *C*_0_ = 10 mg/L)

The course of the concentration shows a rapid decrease in the DCF concentration when using a Rh-HK catalyst. In the case of DCF, the degradation rate can be increased by increasing the mass concentration of the catalyst. A Ru hydrogenation catalyst is significantly less reactive under the chosen reaction conditions (Fig S2 (supplementary information)). Buffering the experiment with an acetic acid-acetate buffer (2 mol/L) after 45 min of the experiment running time showed no renewed release of DCF (Fig. 5). Apparently, adsorption does not play a significant role regarding the decrease in the pollutant’s concentrations applying Mg and certain hydrogenation catalysts such as Rh or Pd; that is, the pollutant is actually chemically degraded.

The course of the concentration shows a slight decrease in the IBP concentration (Fig. 4a). The process can be accelerated by applying a larger amount of Rh catalyst. By using the IBP sodium salt, IBP is in a deprotonated form. The carboxylate group present is stabilized via resonance and is more insensitive to reduction. A dehalogenation reaction as in DCF is not possible because the IBP molecule has no halogen atoms. The aromatic moiety of IBP also shows a lower affinity for hydrogenation due to its substituents. The isobutyl moiety/group exerts an + I effect, which increases the electron density in the aromatic system and adsorption to the catalyst surface. This is accompanied by greater stability on the catalyst surface and a lower reaction rate (Stanislaus and Cooper 1994).

EE2 showed a rapid decrease in concentration due to catalytic hydrogenation. EE2 is entirely removed from the sample solution within 20 min (Fig. 4a). The EE2 molecule has an ethynyl group on the C17 atom. C-C triple bonds are usually subject to rapid hydrogenation, which may explain why the EE2 is rapidly degraded. BPA concentration decreases with Rh-HK slower than the EE2 and DCF and faster than the IBP. In contrast to DCF and EE2, only the aromatic systems of BPA can be hydrogenated (Kuklin et al. 2016). After 120 min, BPA was almost completely removed from the solution.

**Fig. 6:**
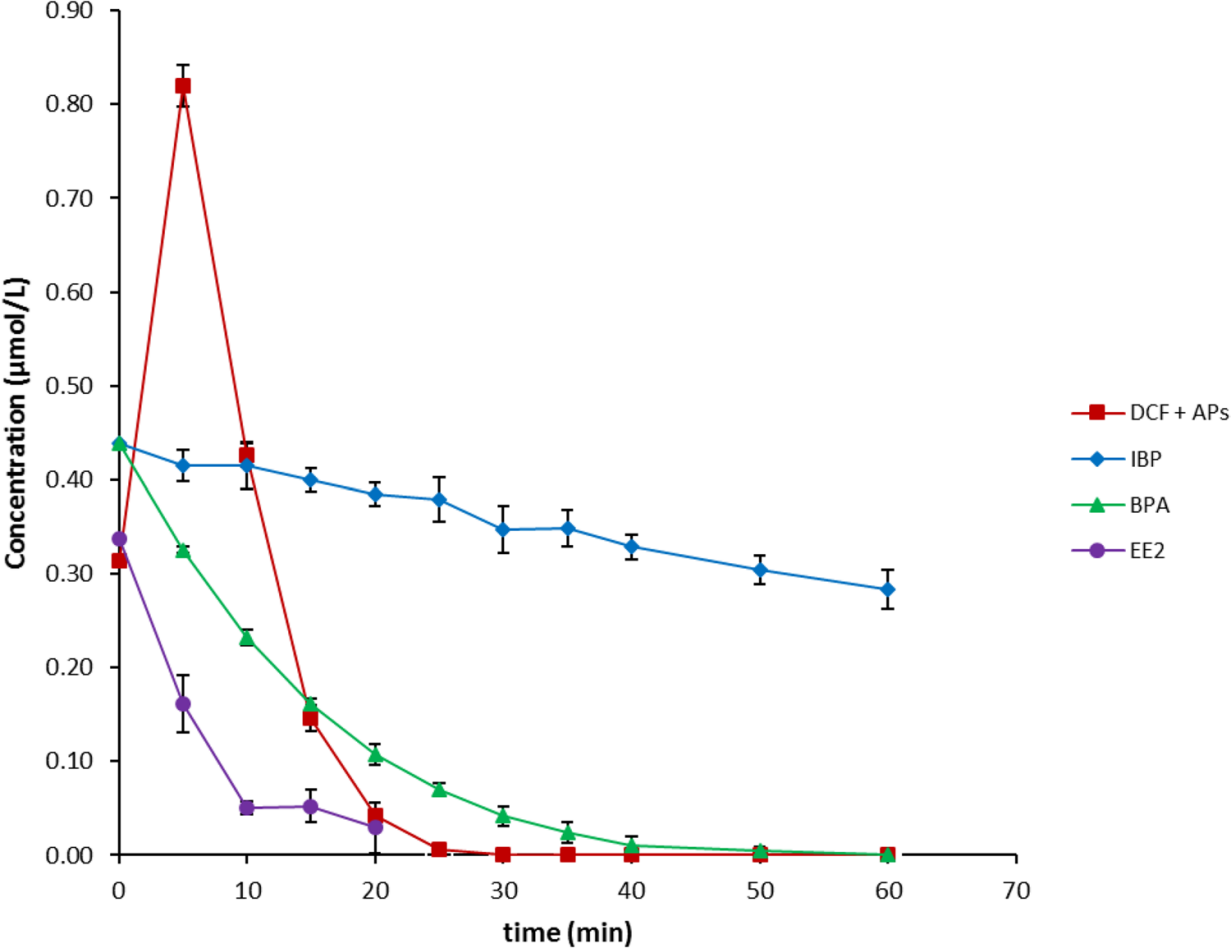
Degradation of DCF, IBP, BPA and EE2 with Mg and Rh-HK at 25 °C (*C*_Mg_ = 25 g/L; *C*_Rh-HK_ = 1,000 mg/L; *C*_0,__pollutant_ = 100 µg/L). “APs” denotes degradation product(s) of DCF – in the early stage of the reaction the first degradation product(s) appear in the LC-MS-MS chromatogram at the same retention time as the parent DCF peak, thus, leading to an increase in the total area of those overlapping peaks

The initial concentration of the model pollutants in the pollutant mixture was 100 µg/L each. Tests for the sample substance mixture degradation were also carried out at magnesium mass concentrations of 25 g/L and Rh-HK mass concentrations of 1,000 mg/L. EE2, BPA, and DCF and their associated degradation products (APs) were completely removed from the pollutant solution after 60 min (Fig. 6). Only IBP turned out to be slowly/poorly degradable. After 1 h, the concentration curve for IBP shows a decrease to about 65 % in the initial concentration. Note that, the degradation curve of DCF did not reflect the real course of its concentration. The separation in the HPLC was insufficient so that the peaks of DCF and its first degradation products (APs) probably overlapped. The overlapping of peaks having virtually the same retention time led to an (of course feigned, pretended) increase in the degradation curve. Thus, applying the DCF calibration to the entire peak led to an apparent error that was difficult to calculate since the UV absorption behavior of the DCF and the APs cannot be assumed to be the same. A quantitative separation and evaluation were not possible (in this initial phase of the reaction). After the initial phase of the reaction, however, neither DCF nor UV-active APs could be detected in the sample solution any longer regarding these experiments using the UV detector only.

### Degradation with Mg and Pd-HK

**Fig. 7:**
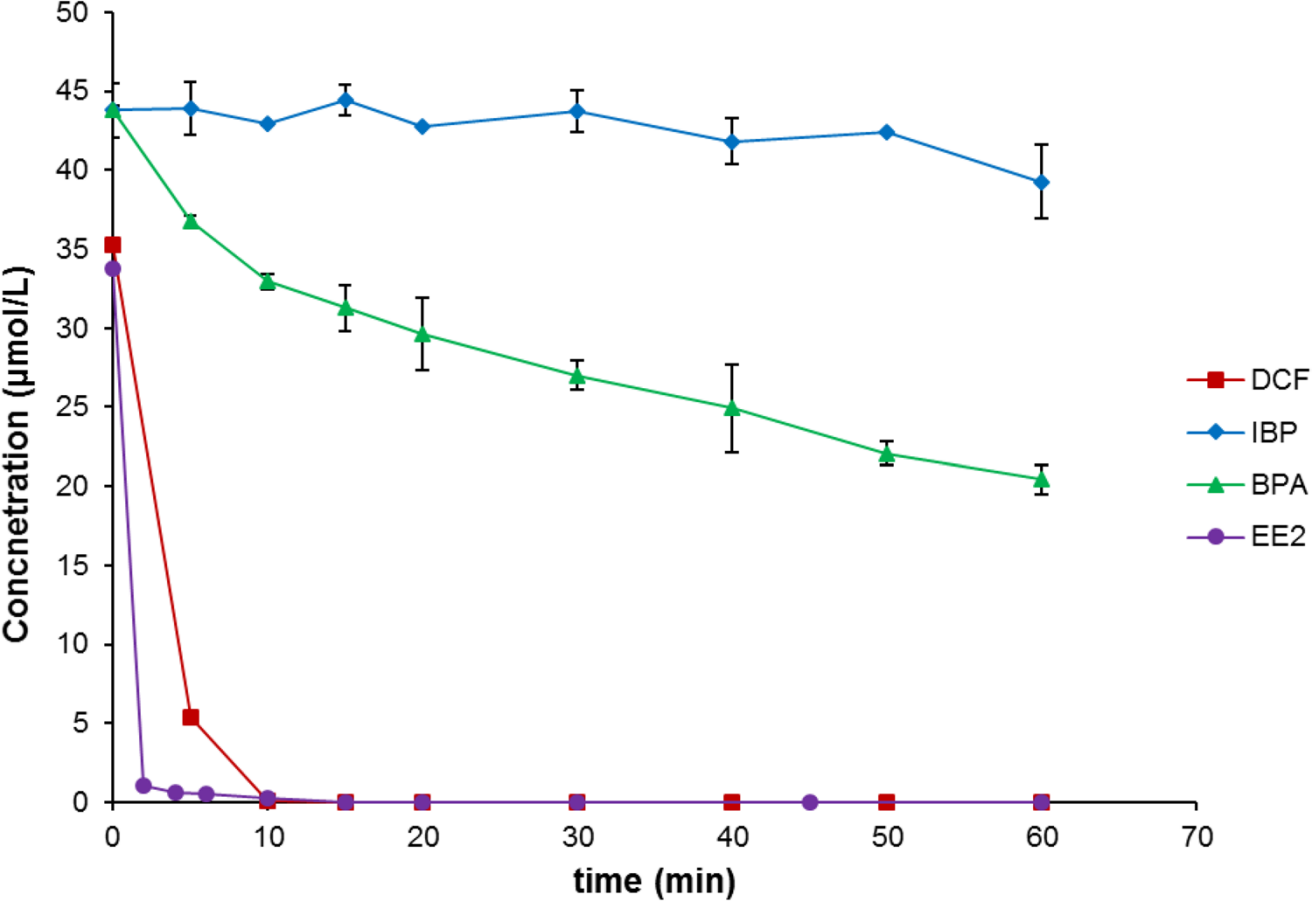
Degradation of DCF, IBP, BPA and EE2 (single substance) with Mg and Pd-HK at 25 °C (*C*_Mg_ = 25 g/L; *C*_Pd-HK_ = 1,030 mg/L; *C*_0,__pollutant_ = 10 mg/L)

The concentrations of DCF and EE2 decreased faster on Pd-HK than on Rh-HK. However, IBP showed similar behavior on both catalysts after 60 min (very slow/poor degradation). On the other hand, BPA was removed more quickly from the solution when employing the Rh-HK. In the case of DCF, chromatograms showed a single peak of an AP. It is reasonable to conclude that it is the product of hydrodechlorination (Zhu and Lim 2007). In the reaction of DCF when applying the Pd-HK, hydrodechlorination is described as the only reaction mechanism that occurs (De Corte et al. 2012). Figure 7 summarizes the breakdown of the individual sample substances at a mass concentration of Pd-HK of 1,030 mg/L. DCF and EE2 degraded quickly and were no longer detectable after 10 and 4 min, respectively. Slow decreases in concentration were observed for BPA and especially for IBP. After 60 min, the BPA concentration was around 28 µmol/L, which was 65 % of the initial concentration. After 60 min, the IBP concentration was about 39 µmol/L, which was 90 % of the initial concentration. Moreover, the successful degradation of a pollutant mixture highlights the potential of this approach for comprehensive water treatment, although challenges in quantifying the degradation of individual pollutants within complex mixtures remain. Overall, these findings strikingly revealed the efficacy of reactive zero-valent metal systems (covering certain base metals, that is, ZVM, being even more reactive rather than ZVI) coupled with hydrogenation catalysts for efficient pharmaceutical degradation in water.

### Degradation products (APs)

**Fig. 8:**
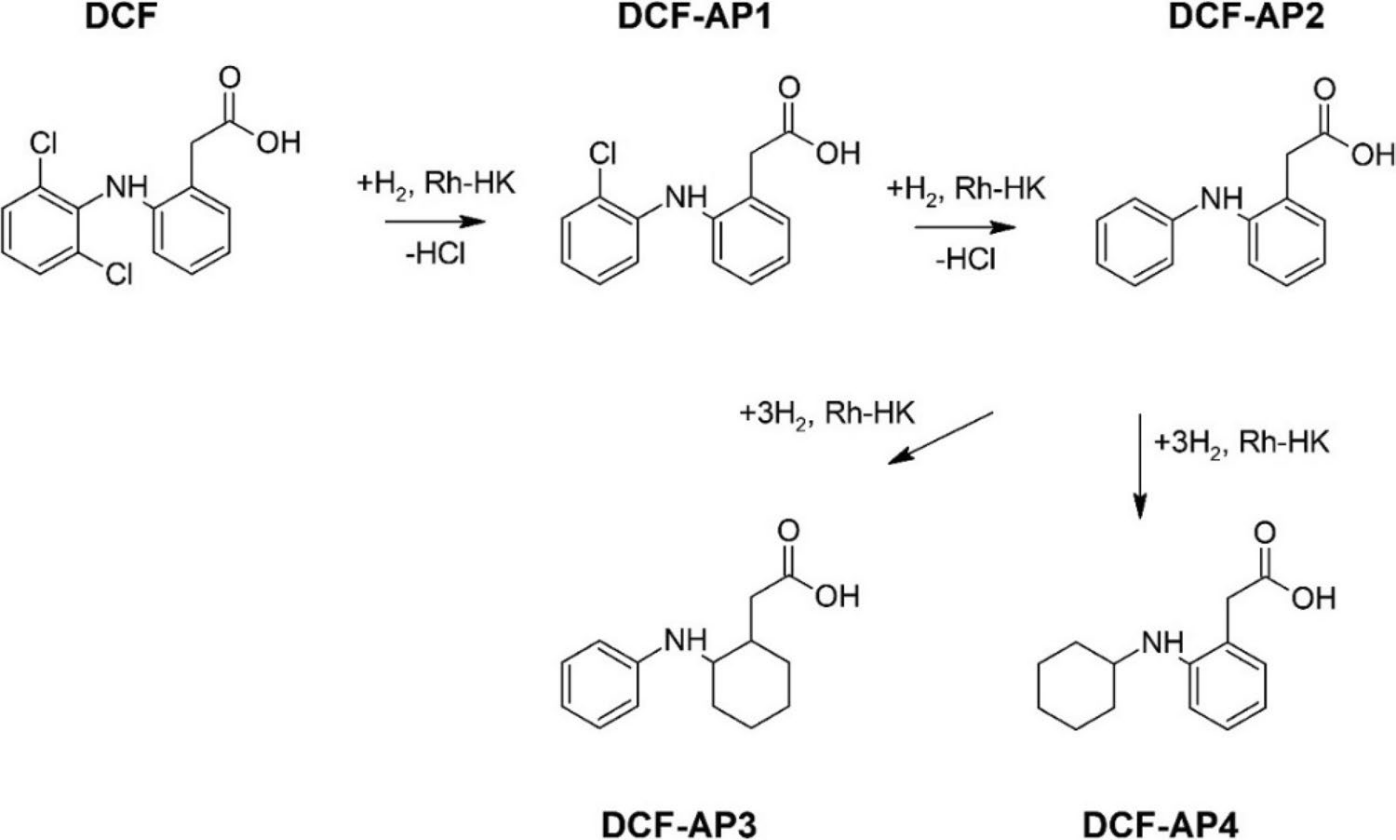
Degradation products and assumed pathways of the degradation of DCF by catalytic dehydodehalogenation and hydrogenation employing Rh-HK in water

The MS spectra of all degradation products of DCF were examined and their respective retention times (R_t_(s)), as shown in the HPLC, are compiled in Table 3. The hypothetical reaction mechanisms comprising two consecutive hydrodehalogenation (hydrodechlorination) steps followed by aromatic hydrogenation are shown in Fig. 8. With the help of the monoisotopic masses (M_mi_), the m/z ratios, the R_t_, the isotopic patterns, the fragment ion peaks which occur, and an MS database (Patiny and Borel 2013), the formulation of suspected degradation products could actually be verified. The plausibility of the molecular formula was checked using reference spectra stored in databases and a comparison of the characteristic isotope patterns. Any fragment peak what occurred provided certain information on functional groups. DCF (R_t_ = 7.13 min) has a molecular ion at m/z 294 with a fragment ion at m/z 250. The first degradation product DCF-AP1 (R_t_ = 6.97 min) could be identified as a singly dechlorinated DCF derivative. It has a molecular ion at m/z 260 with a fragment ion at m/z 216. DCF-AP2 (R_t_ = 6.54 min) has a molecular ion at m/z 226 with a fragment ion at m/z 182. The isotope pattern here actually confirms the absence of both chlorine atoms. DCF was used as its sodium salt, in other words, the carboxyl group of DCF is present as a carboxylate group. The carboxylate group is resonance stabilized and should be relatively inert to hydrogenation (reduction). One reliable explanation for the rapid degradation of DCF is the course of the hydrodechlorination reactions. DCF-AP3 and DCF-AP4 (R_t_ = 5.53 min) show a molecular ion at m/z 232 with a fragment ion at m/z 188, that is, clearly a hydrogenation of the aromatic moieties each took place. This is supported by the difference in the m/z ratios of DCF-AP2 and DCF-AP3 or DCF-AP4, which results from the addition of hydrogen atoms. DCF-AP3 and DCF-AP4 cannot be distinguished using MS. The dechlorinated intermediate products DCF-AP1 and DCF-AP2 can be detected first on the basis of the recorded chromatograms. In the first step, a single dechlorinated DCF derivative is formed, which is dechlorinated again in the second step (Buil et al. 2010; De Corte et al. 2012). Furthermore, regarding BPA (R_t_ = 6.24 min), it has a molecular ion at m/z 227. The degradation product BPA-AP (R_t_ = 6.35 min) with a molecular ion at m/z 239 suggests a fully hydrogenated BPA derivate.

**Table 3:**
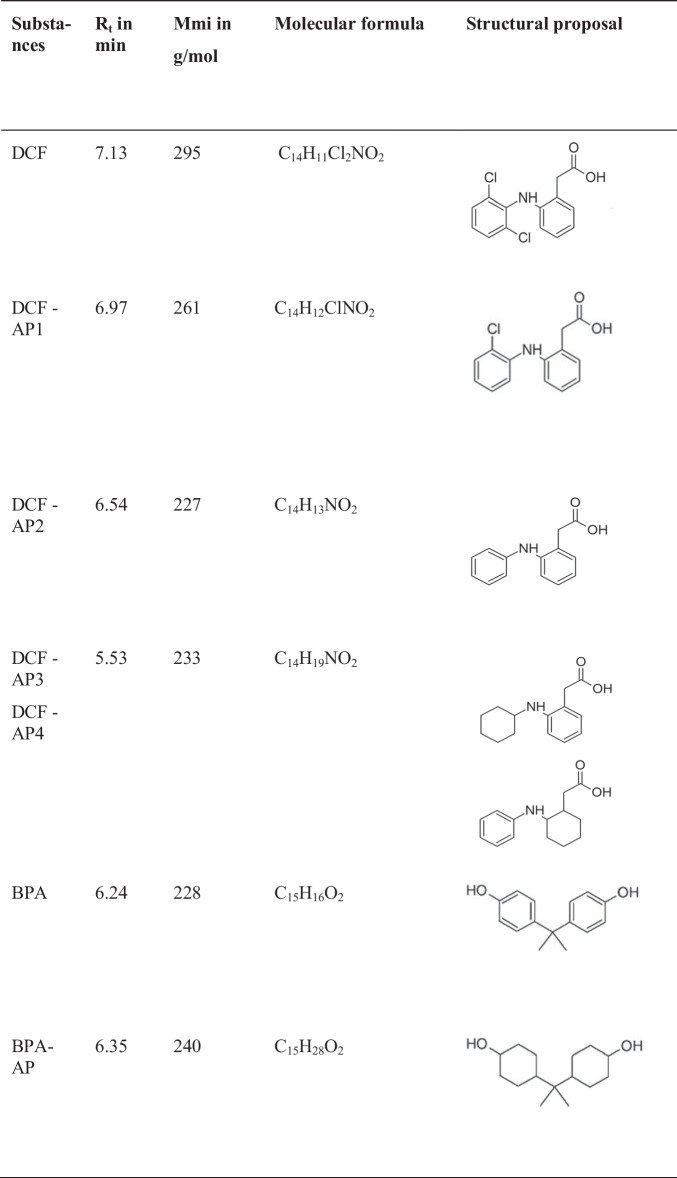
Reaction products of the catalytic hydrogenation of DCF and BPA with Rh-HK

### Kinetics of catalytic hydrodehalogenation and hydrogenation

**Fig. 9:**
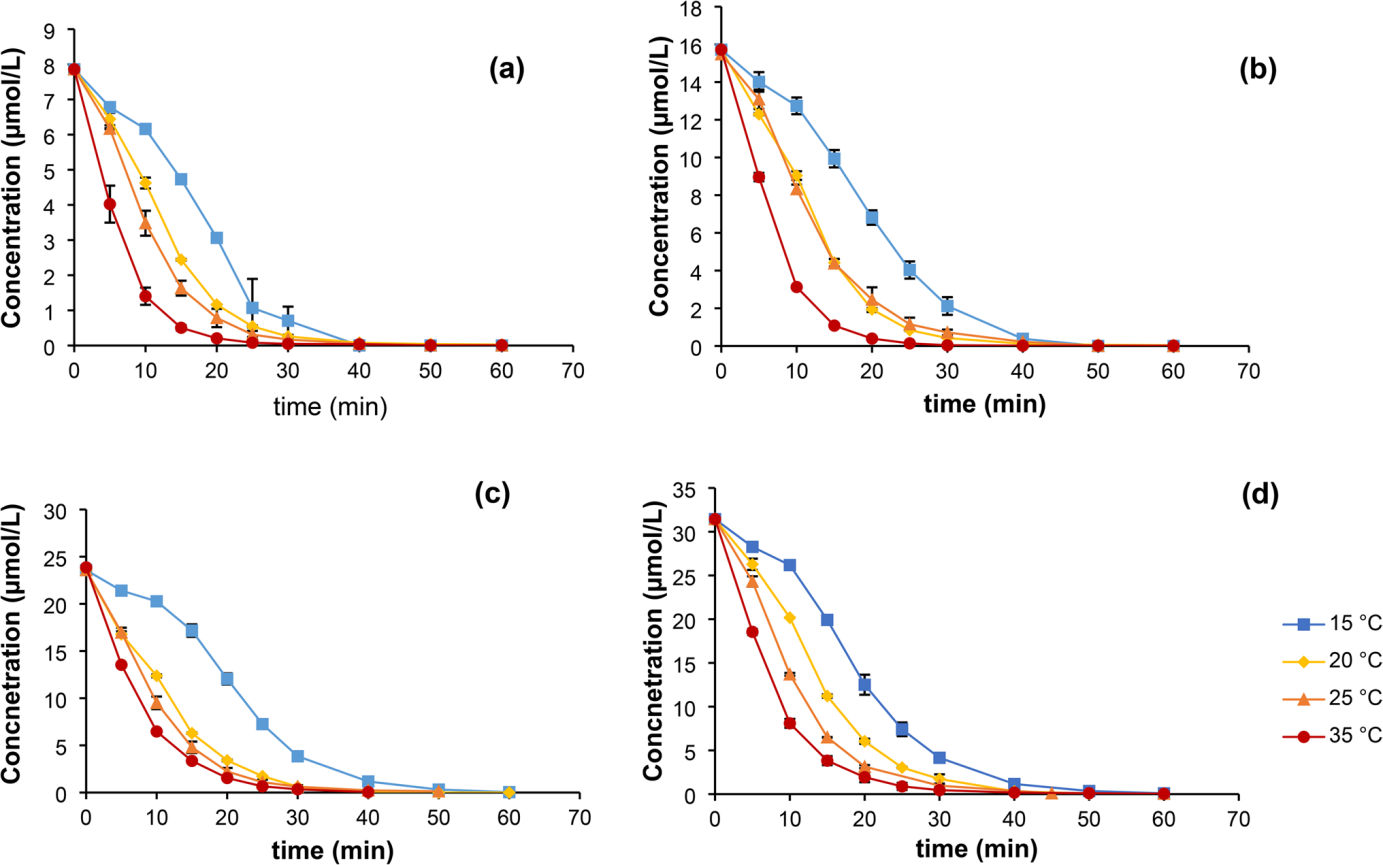
Concentration curves vs time for DCF degradation with Mg (25 g/L) and Rh-HK (1,000 mg/L) at four different temperatures, i.e., 15 °C, 20 °C, 25 °C, and 35°; **a** DCF initial concentration *C*_0_ = 7.86 µmol/L (2.5 mg/L), **b** DCF initial concentration *C*_0_ = 15.72 µmol/L (5.0 mg/L), **c** DCF initial concentration *C*_0_ = 23.57 µmol/L (7.5 mg/L), and **d** DCF initial concentration *C*_0_ = 31.43 µmol/L (10.0 mg/L)

**Fig. 10:**
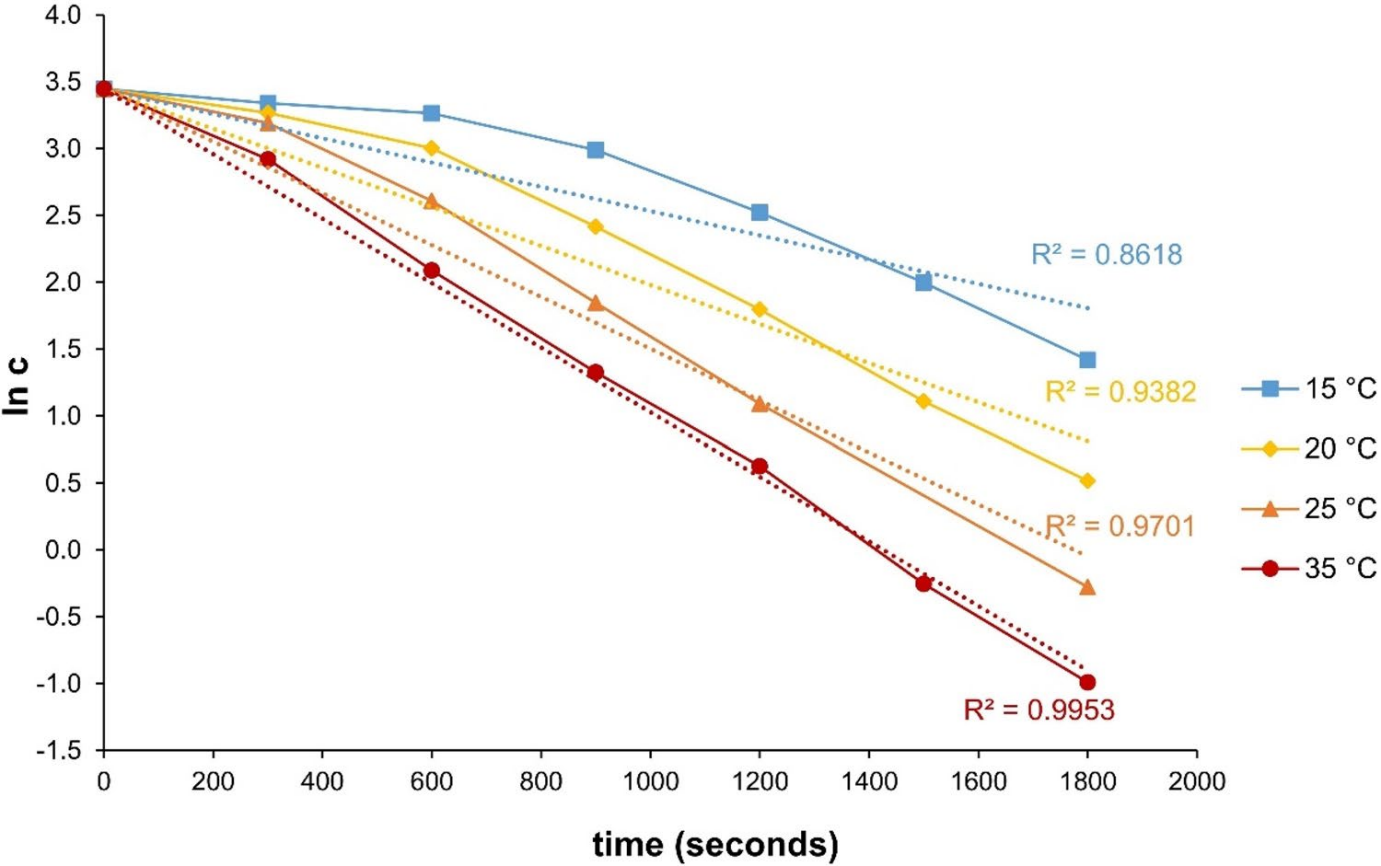
Plots of ln c (DCF) versus t at different temperatures (*C*_Mg_ = 25 g/L; *C*_Rh-HK_ = 1,000 mg/L; *C*_0_ = 31.43 µmol/L (10 mg/L)) showing a poor linear correlation due to initial induction (lag) periods (except at 35 °C)

The degradation of DCF with Rh-HK and Mg powder activated with ammonium chloride was investigated at different temperatures. In order to generate a sufficient number of data sets for modelling, the degradation tests described above were carried out at temperatures of 15 °C, 20 °C, 25 °C, and 35 °C. The initial concentrations of DCF were also varied, that is, 2.5 mg/L (7.86 µmol/L), 5.0 mg/L (15.72 µmol/L), 7.5 mg/L (23.57 µmol/L), and 10.0 mg/L (31.43 µmol/L). The different concentration curves at the different temperatures are shown in Fig. 9. Further, Fig. 10 shows plots of ln(*c*) versus time. If the reaction follows a first-order rate law (Eq. 7), the plot should result in a straight line with a slope ${k}_{\mathrm{\vartheta }}$. It can be seen that this linear relationship can be verified at a temperature of 35 °C only due to a regression coefficient of determination *R*^2^ of 0.996 (Baena-Nogueras et al. 2017).

7 $$\frac{d{c}_{DCF}}{dt}=-{k}_{\vartheta }\cdot {c}_{DCF}$$

For lower temperatures, the values for *R*^2^ become significantly smaller. Degradation runs at lower temperatures, especially such as 15 °C or 20 °C, show a non-linear course pertaining to a ln *c* versus time plot. In the beginning, there is a significant induction (lag) phase of the reaction may be due to the necessary development of constant reaction conditions (full reactivity of hydrogen at the catalyst's surface is not reached over the initial course of the reaction, being pretty typical for heterogeneous reactions in solution). Moreover, also due to at the beginning of the activation of the Mg (surface) by ammonium chloride, a full reactive surface is formed not until a constant reactivity is finally reached (after some minutes in this case to be seen by analysing the experimental data). As the temperature increases, however, the impact of the induction phase on the overall reaction rate decreases. A reaction between activated hydrogen and oxygen dissolved in the water may also be conceivable (Suppiah et al. 1988; Shi et al. 2009). Consequently, until the dissolved oxygen has not quickly been removed yet, the hydrogen is not available or only available to a limited extent for actual catalytic hydrogenation.

In order to take these effects into account, following Birke et al. (2011), an extension of the first-order rate law by a time-dependent "surface function" *S*(*t*) (Eq. 8), which represents a logistic growth function simulating the development of reactive sites/activation of molecular hydrogen on the Rh-HK surface for reductive dechlorination and hydrogenation of DCF over time is introduced. It’s particularly assumed that in this initial reaction phase – especially at lower temperatures – the degradation of DCF is lowered by an induction (lag) phase, wherein the full reactivity of molecular hydrogen has not been reached yet. Note that, *k*, *k*_1_, and *k*_2_ are special rate constants in this individually set up kinetic model regarding heterogenous degradation reactions of pollutants (Birke et al., 2011). Similar models have been developed recently, too (Baena-Nogueras et al. 2017).

8 $$S(t)=\frac{k}{1+{e}^{(-\hspace{0.33em}{k}_{1}(t\hspace{0.33em}-\hspace{0.33em}{k}_{2}))}}$$

The introduction of S(t) results in a modified rate law for Eq. 7, see Eq. 9.

9 $$\frac{d{c}_{DCF}}{dt}=-\hspace{0.33em}{k}_{\vartheta }\cdot \hspace{0.33em}{c}_{DCF}\cdot \hspace{0.33em}S(t)=-\hspace{0.33em}{k}_{\vartheta }\cdot \hspace{0.33em}{c}_{DCF}\cdot \hspace{0.33em}\frac{k}{1+{e}^{(-\hspace{0.33em}{k}_{1}(t\hspace{0.33em}-\hspace{0.33em}{k}_{2}))}}$$

Regarding different temperatures, one thus obtains a system of ordinary differential equations (ODE) shown in Eqs. 10–13 regarding different isothermal conditions (reaction Celsius temperatures ϑ applied between 15 ° and 35 °C, of course recalculated in absolute temperatures in K).

10 $$\frac{d{c}_{DCF}}{dt}=-\hspace{0.33em}{k}_{35}\cdot \hspace{0.33em}{c}_{DCF}\cdot \hspace{0.33em}S(t)$$

11 $$\frac{d{c}_{DCF}}{dt}=-\hspace{0.33em}{k}_{25}\cdot \hspace{0.33em}{c}_{DCF}\cdot \hspace{0.33em}S(t)$$

12 $$\frac{d{c}_{DCF}}{dt}=-\hspace{0.33em}{k}_{20}\cdot \hspace{0.33em}{c}_{DCF}\cdot \hspace{0.33em}S(t)$$

13 $$\frac{d{c}_{DCF}}{dt}=-\hspace{0.33em}{k}_{15}\cdot \hspace{0.33em}{c}_{DCF}\cdot \hspace{0.33em}S(t)$$

**Fig. 11:**
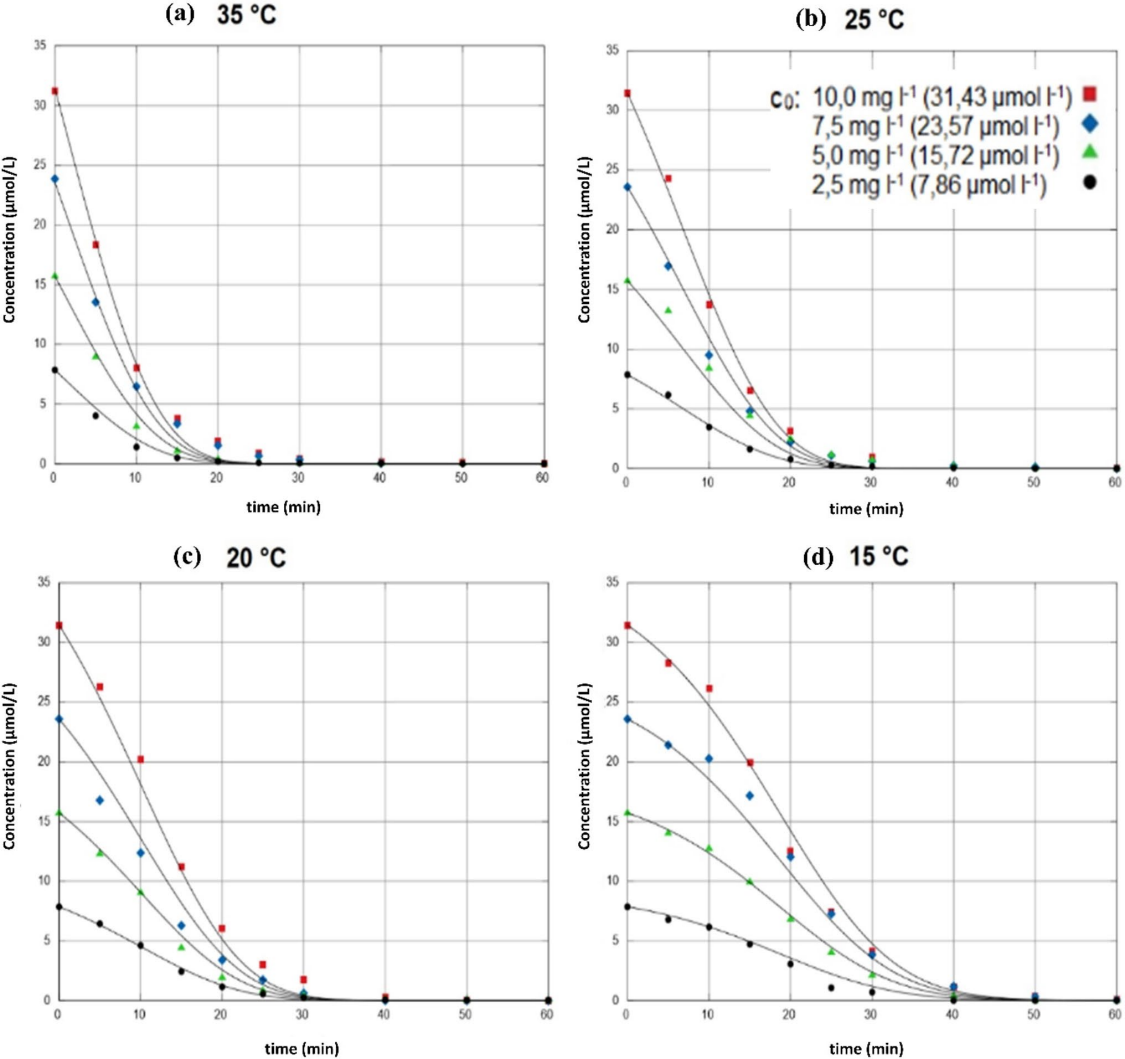
Simultaneous fits of the datasets for DCF degradation at four different concentrations (7.86 µmol/L, 15.72 µmol/L, 23.57 µmol/L, and 31.43 µmol/L) and at four different temperatures (15 °C, 20 °C, 25 °C, and 35 °C) each to the ODE system of rate equations (Eqs. 10–13) using EASY-FIT model design software

Using this ODE system, the data sets were plotted to the associated model underpinned using EASY-FIT model design software (Schittkowski 2002, 2019). The graphic results of the simultaneous fits are shown in Fig. 11. The simultaneously calculated rate constants *k*_*ϑ*_ associated with the respective temperatures are listed in Table 4.

**Table 4:** Rate constants were determined using EASY-FIT model design software for parameter estimation of the Arrhenius equation (activation energy)

*ϑ* in °C	*T* in K	1/T in 1/K	*k*_*ϑ*_ in 1/min	*k*_*ϑ*_ in 1/s
15	288	0.00347	0.2885	0.0048
20	293	0.00341	0.6590	0.0110
25	298	0.00336	0.9201	0.0153
35	308	0.00325	1.5881	0.0265

**Fig. 12:**
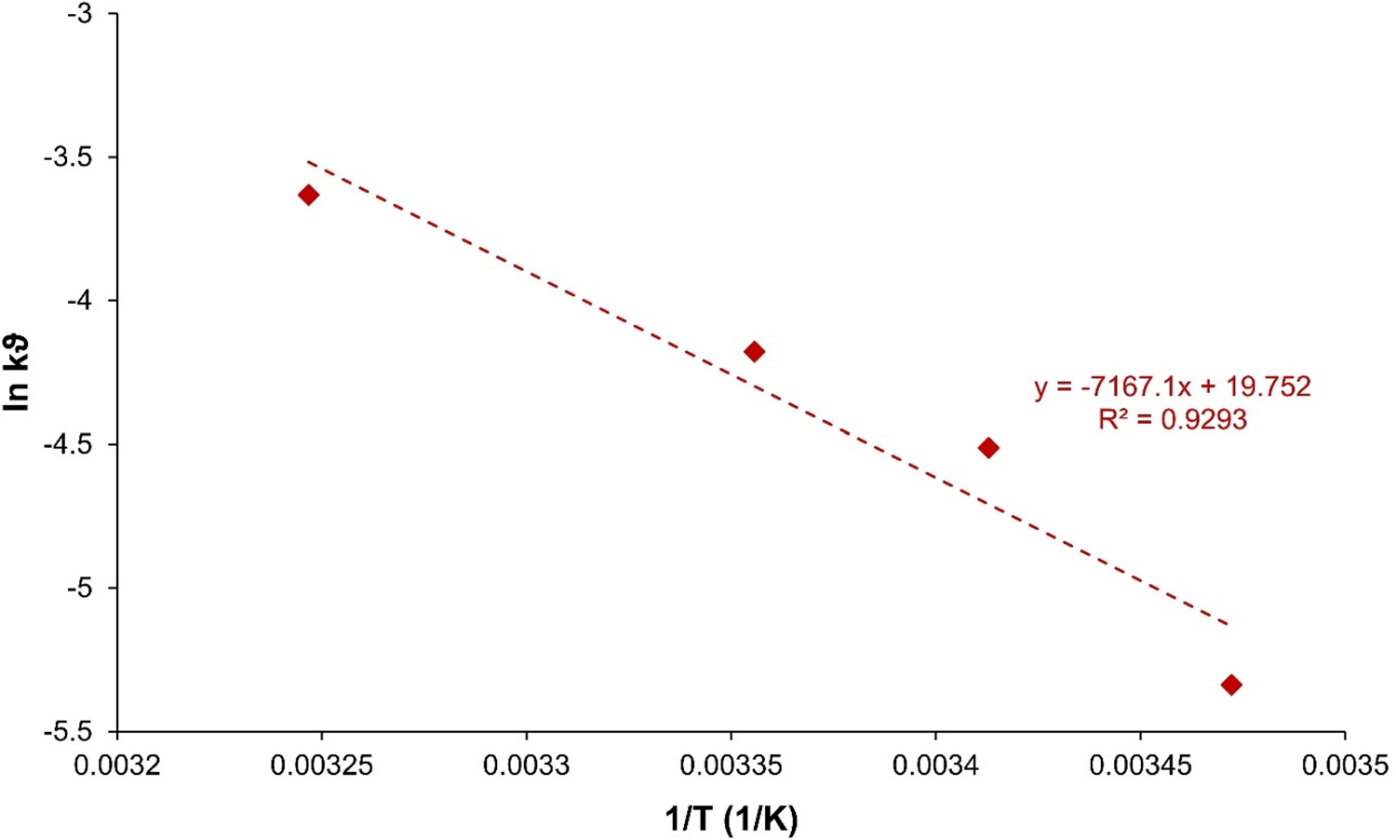
Arrhenius plot of ln k_ϑ_ values against 1/T

It can be seen that these simultaneous (kinetic) plots mirror the course of the measured experimental values very well. Hence, the applied mathematical model with the selected *S*(*t*) term is suitable for fitting the measured real data to the kinetic model. From the temperature-dependent rate constants determined in EASY-FIT, ln *k*_*ϑ*_ was plotted against 1/*T* (Eq. 14, Fig. 12). Since the fit of the values to a straight line shows a good correlation, the reaction behavior can be described using the well-known Arrhenius equation.

14 $${k}_{\vartheta }=A\cdot {e}^{-\hspace{0.33em}\frac{{E}_{A}}{RT}}$$

The line of best fit has the slope $-{E}_{A}/R$. The activation energy *E*_*A*_ could be calculated from Eq. 15.

15 $${E}_{A}=-R\cdot (-{7,167}.1 {\text{K}})=8.314 {{\text{JK}}}^{-1}{{\text{mol}}}^{-1}\cdot {7,167}.1 {\text{K}}={59}.6 {{\text{kJmol}}}^{-1}$$

Table S5 (supplementary information) shows a selection of literature values for the activation energies of related reactions. Note that, the determined value for *E*_*A*_ of 59.6 kJ/mol is in pretty good accordance with some literature values. In other words, the activation energies of heterogeneously catalyzed reactions are in the range of 50 to 100 kJ/mol. This confirms the plausibility of the determined value for *E*_*A*_. Note that in the case of the reaction of DCF on Rh-HK, no distinction was made between hydrodechlorination and catalytic hydrogenation when calculating the *E*_*A*_ value. Hence, it might remain a rough estimation. Both possible hydrodechlorination reactions (chlorine atom 1 and 2) were recorded in total, however. Further series of measurements, in which temperature and initial concentrations should be varied, could statistically further secure the determination of the activation energy.

## Conclusions

The findings of this study emphasize the limited efficacy of iron as well as higher reactive magnesium metal as a single reagent (the only reagent alone) each, that is, without additives, in removing pharmaceutical pollutants, such as DCF and BPA, from water. However, the utilization of supported and unsupported iron bimetals exhibits a considerable attenuation in the concentration of the pollutants through a combination of degradation and adsorption mechanism, that is, on the metals' surface and/or presumably by adsorption on hydroxides, respectively. The outcomes demonstrated that DCF is removed over 90 % when exposed to Fe-Cu or Fe-Ni bimetals as single substance. However, mixture substances have lowered the yield of degradation reaction as it decreased the efficacy of DCF to 73 % and 23 % with Fe-Cu and Fe-Ni respectively. Notably, metal corrosion during the reaction can induce structural altercation in iron particles, potentially releasing toxicologically concerning non-ferrous metals like Cu or Ni. Consequently, future investigations must delve into a “real world” aqueous matrix to accurately assess pollutant removal efficacy and explore the feasibility of iron particle regeneration. In contrast, a combination of Mg and hydrogenation catalysts such as Rh emerges as a noteworthy avenue for effectively degrading pharmaceuticals and other persistent pollutants such as DCF and BPA through heterogeneous catalytic hydrogenation.

Mg in combination with Rh-HK showed rapid degradation of DCF, EE2, and BPA concentrations up to approx. 100 % in a short time. By converting the aromatic systems of pharmaceutical substances into cyclic alkanes or alkenes and, moreover, when halogen atoms are attached to the molecule as in DCF, by dehalogenation, catalytic hydrogenation can help improve the degradation of persistent drug residues significantly. Note that various structural elements can be broken down or converted by means of catalytic hydrogenation. Therefore, the application of bimetal reactions, mainly of Mg and noble metals, offers a compelling strategy to rapid degradation of toxic pharmaceuticals and other organic pollutants. Thus, cyclohexyl derivatives generated by (green) hydrogen from the aromatic systems of the pollutants should be less toxic and/or could be expected to be more readily degradable in sewage, that is, in combination with ozonation and/or microbial degradation. This is maybe a brand-new re-evaluation for the development of promising, highly innovative future applications to these currently absolutely urging environmental and health issues, that is, globally employing green hydrogen to destroy abundant water pollutants more sustainably!

### Supplementary Information

Below is the link to the electronic supplementary material.

Supplementary file1 (DOCX 415 KB)

## Data Availability

All data generated or analyzed during this study are included in this article.
