# Supplementary material for: Degradation of pharmaceuticals and other emerging pollutants employing bi-metal catalysts/magnesium and/or (green) hydrogen in aqueous solution
Source: Environ Sci Pollut Res Int. 2024 May 15;31(24):35992–6012. doi: 10.1007/s11356-024-32777-1 (PMC11136818; doi:10.1007/s11356-024-32777-1)
Supplement: Supplementary file 1 — Supplementary file1 (DOCX 415 KB) [file 11356_2024_32777_MOESM1_ESM.docx]

***Supplementary Information***

**Degradation of pharmaceuticals and other emerging pollutants employing bi-metal catalysts / magnesium and / or (green) hydrogen in aqueous solution**

**Volker Birke, Rahul Singh*, Oliver Prang**

Hochschule Wismar – University of Applied Sciences, Technology, Business and Design, Department of Mechanical Engineering / Process and Environmental Engineering, Philipp-Müller-Str. 14, 23966 Wismar, Germany

Email - [volker.birke@hs-wismar.de](mailto:volker.birke@hs-wismar.de); [rahul.singh@hs-wismar.de](mailto:rahul.singh@hs-wismar.de); oprang@gmx.net

*Corresponding Author:

Rahul Singh

Research Associate,

Department of Mechanical Engineering / Process and Environmental Engineering,

Hochschule Wismar – University of Applied Sciences, Technology, Business and Design, Philipp-Müller-Str. 14, 23966 Wismar, Germany

Email – [rahul.singh@hs-wismar.de](mailto:rahul.singh@hs-wismar.de)

Phone – +49-1744304988

**Table S1** List of chemicals and materials used, along with the manufacturer details, during the experimental process

| **Material** | **Manufacturer** | **Quality/Comment** |
| --- | --- | --- |
| Acetone | Chemsolute | ≥ 99 % |
| Acetonitrile (HPLC Grade) | Merck | Gradient grade for LC |
| Formic acid | AppliChem | 98 % |
| ammonium chloride | Neo Lab Migge | ≥ 99.5 % |
| Bisphenol A | Sigma Aldrich | ≥ 99 % |
| Diclofenac sodium salt | Alfa Aesar | ≥ 98 % |
| Iron (ZVI) | Würth iron works | ≥ 94 % |
| Acetic acid | Chemsolute | ≥ 99 % |
| Ethinyl estradiol | HPC | ≥ 99.7 % |
| Ibuprofen sodium salt | Sigma Aldrich | ≥ 98 % |
| Copper(II) chloride | Merck | for analysis |
| Copper(II) nitrate trihydrate | Carl Roth | ≥ 99 % |
| Magnesium (ZVM) powder | Merck | ≥ 97 % |
| Sodium acetate | Chemsolute | ≥ 99 % |
| Nickel(II) nitrate hexahydrate | Carl Roth | ≥ 99 % |
| Palladium hydrogenation catalyst | Sigma Aldrich | 5 % Pd on Al_2_O_3_ |
| Rhodium hydrogenation catalyst | Sigma Aldrich | 5 % Rh on Al_2_O_3_ |
| Ruthenium hydrogenation catalyst | Sigma Aldrich | 5 % Ru on Al_2_O_3_ |
| Hydrochloric acid | Chemsolute | 32 % |
| Water for HPLC | Merck | LC-MS grade |
|  |  |  |

**Table S2** Redox Potential of Various Noble Metals Used as Bimetal with ZVI

| **M^n+^/M** | **Reaction** | **E^0^ in V** | **Salts** |
| --- | --- | --- | --- |
| Cu^II^/Cu^0^ | $Cu^{2+}+ 2 e^{-} \xrightleftharpoons{} Cu$ | 0.339 | Cu(NO_3_)_2_ ∙ 3 H_2_O |
|  |  |  | CuCl_2_ ∙ 2 H_2_O |
| Ni^II^/Ni^0^ | $Ni^{2+}+ 2 e^{-} \xrightleftharpoons{} Ni$ | -0.236 | Ni(NO_3_)_2_ ∙ 6 H_2_O |
| In^III^/In^0^ | $In^{3+}+ 3 e^{-} \xrightleftharpoons{} In$ | -0.338 | In(III)-acetate |
| Sn^II^/Sn^0^ | $Sn^{2+}+ 2 e^{-} \xrightleftharpoons{} Sn$ | -0.141 | SnCl_2_ ∙ 2 H_2_O |
| Co^II^/Co^0^ | $Co^{2+}+ 2 e^{-} \xrightleftharpoons{} Co$ | -0.282 | Co(NO_3_)_2_ ∙ 6 H_2_O |
| Fe^II^/Fe^0^ | $Fe^{2+}+ 2 e^{-} \xrightleftharpoons{} Fe$ | -0.440 | - |

**Table S3** Details of all the parameters of the HPLC methods used.

|  | **ME1** | | | **ME2** | | | **ME3** | | |
| --- | --- | --- | --- | --- | --- | --- | --- | --- | --- |
| **Pillar** | **Dr. Maisch Reprosil Pur 120 C18-AQ** | | | **Dr. Maisch Reprosil Pur 120 C18-AQ** | | | **Agilent ZORBAX Eclipse Plus C18** | | |
| Column length | 50 mm | | | 50 mm | | | 250 mm | | |
| Column diameter | 2 mm | | | 2 mm | | | 4.6 mm | | |
| Pore diameter of the column | 3 µm | | | 3 µm | | | 5 µm | | |
| mobile phase | H_2_O/ACN | | | H_2_O/ACN | | | H_2_O/ACN | | |
| Column oven temperature | 30 ℃ | | | 30 ℃ | | | 40 ℃ | | |
| Duration | 13 mins | | | 9 mins | | | 20 mins | | |
| flow | 0.4 ml/min | | | 0.4 ml/min | | | 0.6 ml/min | | |
| gradient | t in min | H_2_O in % | ACN in % | t in min | H_2_O in % | ACN in % | t in min | H_2_O in % | ACN in % |
|  | 0.0 | 95 | 5 | 0.0 | 95 | 5 | 0.0 | 95 | 5 |
|  | 0.2 | 95 | 5 | 0.2 | 95 | 5 | 0.2 | 95 | 5 |
|  | 7.0 | 0 | 100 | 6.0 | 0 | 100 | 13.0 | 0 | 100 |
|  | 10.0 | 0 | 100 | 7.0 | 0 | 100 | 18.0 | 0 | 100 |
|  | 10.1 | 95 | 5 | 7.1 | 95 | 5 | 18.5 | 95 | 5 |
|  | 13.0 | 95 | 5 | 9.0 | 95 | 5 | 20.0 | 95 | 5 |
| Injected sample volume | 10 µl | | | 10 µl | | | 100 µl | | |

Table S4 Parameters for MS-Method.

| **Source** | | **Analyser** | |
| --- | --- | --- | --- |
| Parameter | Value | Parameter | Value |
| Capillary | 2,50 | LM Res 1 | 15,0 |
| Cone | 19 | HM Res 1 | 15,0 |
| Extractor | 2 | IEnergy 1 | 0,5 |
| RF Lense | 1,00 | Entrance | 50 |
| Source Block Temperature | 120 | Collision | 2 |
| Desolvation Temperature | 350 | Exit | 50 |
|  |  | LM Res 2 | 15,0 |
|  |  | HM Res 2 | 15,0 |
|  |  | IEnergy 2 | 3,0 |
|  |  | Multiplier | 700 |

**Table S5** Activation energies for different organic products for catalytic hydrogenation and hydrodechlorination as mentioned in various literature.

| **Catalyst** | **Product** | **E_A_ in kJ/mol** | **References** |
| --- | --- | --- | --- |
| **Catalytic Hydrogenation** | | | |
| Rh, Ru, or Pt on Al_2_O_3_ | benzene | 50.2 | (Amano and Parravano, 1957) |
| Pd on Al_2_O_3_, TiO_2_ or SiO_2_ | benzene | 36.0 to 59.0 | (Chou and Vannice, 1987) |
| Pd on Al_2_O_3_ | benzene, toluene, m-xylene, o-xylene | 49.0 to 62.0 | (Rahaman and Vannice, 1991) |
| **Hydrodechlorination** | | | |
| Pd on ZVI | tetrachloroethene | 31.1 | (Lien and Zhang, 2007) |
| Pd on ZVI | hexachlorobutadiene | 47.1 | (Rodrigues et al., 2017) |
| Ni on Al_2_O_3_ | chlorobenzene | 75.5 | (Keane and Larsson, 2008) |

***References***

Amano A, Parravano G, (1957) The vapor-phase hydrogenation of benzene on ruthenium rhodium, palladium, and platinum catalysts. Adv. Catal. 9: 716–726. <https://doi.org/10.1016/S0360-0564(08)60224-2>

Chou P, Vannice MA (1987) Benzene hydrogenation over supported and unsupported palladium. I. Kinetic behavior. J. Catal. 107: 129–139. <https://doi.org/10.1016/0021-9517(87)90278-8>

Keane MA, Larsson R (2008) On the stepwise change of activation energies in the hydrodechlorination of chlorobenzene over supported nickel. Catal. Commun. 9: 333–336. <https://doi.org/10.1016/j.catcom.2007.06.024>

Lien HL, Zhang WX (2007) Nanoscale Pd/Fe bimetallic particles: Catalytic effects of palladium on hydrodechlorination. Appl. Catal. B Environ. 77: 110–116. <https://doi.org/10.1016/j.apcatb.2007.07.014>

Rahaman VM, Vannice AM (1991) The hydrogenation of toluene and o-, m-, and p-xylene over palladium II. Reaction model. J. Catal. 127: 267–275. <https://doi.org/10.1016/0021-9517(91)90225-S>

Rodrigues R, Betelu S, Colombano S, Masselot G, Tzedakis T, Ignatiadis I (2017) Reductive dechlorination of hexachlorobutadiene by a Pd/Fe microparticle suspension in dissolved lactic acid polymers: Degradation mechanism and kinetics. Ind. Eng. Chem. Res. 56: 12092–12100. <https://doi.org/10.1021/acs.iecr.7b03012>


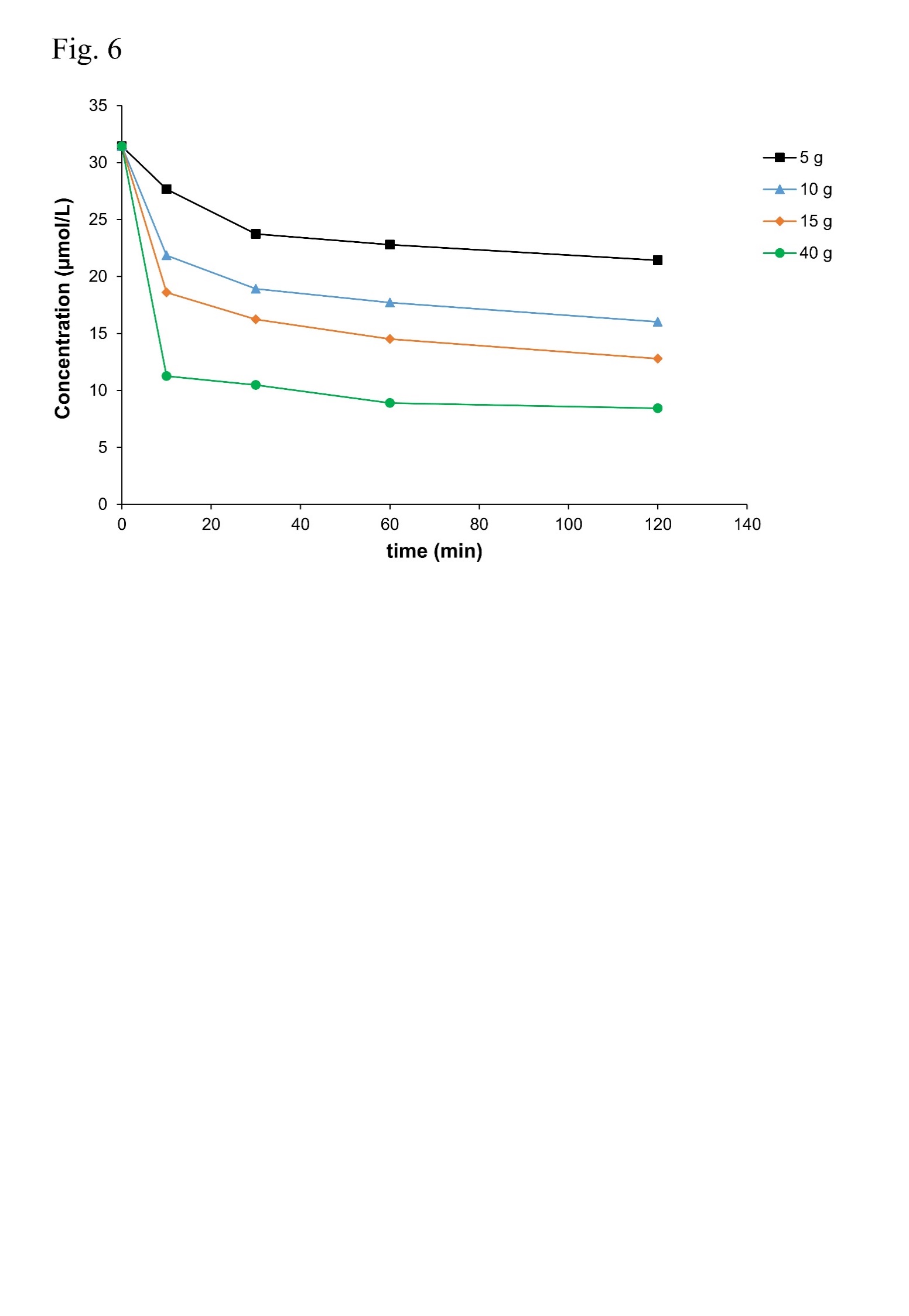


**Fig. S1** Concentration curves for DCF applying different dosages of Mg powder (c_0_,DCF = 31.43 µmol/L; temperature = 25 °C; V = 100 mL)


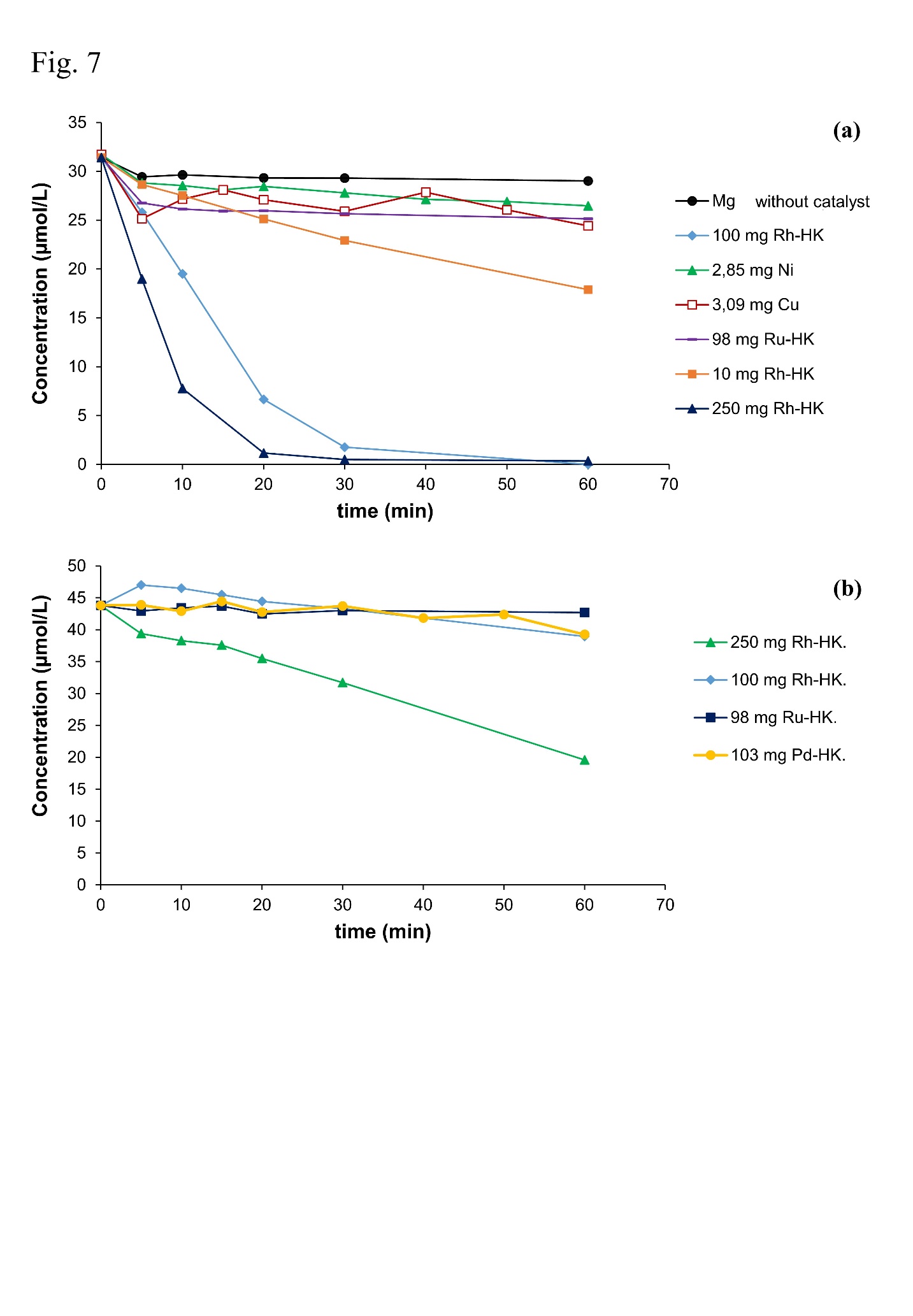


**Fig. S2** Concentration curves for a. DCF degradation with Mg and various catalysts at 25 °C, volume batch = 100 mL (Mg = 2.5 g; C_0,DCF_ = 31.43 mg/L), b. IBP degradation with Mg and HK (Mg = 2.5 g; C_0,IBP_ = 43.81 µmol/L)
